# The Transcriptomes of Two Heritable Cell Types Illuminate the Circuit Governing Their Differentiation

**DOI:** 10.1371/journal.pgen.1001070

**Published:** 2010-08-19

**Authors:** Brian B. Tuch, Quinn M. Mitrovich, Oliver R. Homann, Aaron D. Hernday, Cinna K. Monighetti, Francisco M. De La Vega, Alexander D. Johnson

**Affiliations:** 1 Department of Microbiology and Immunology, University of California San Francisco, San Francisco, California, United States of America; 2 Genetic Systems Division, Research and Development, Life Technologies, Foster City, California, United States of America; 3 Department of Biochemistry and Biophysics, University of California San Francisco, San Francisco, California, United States of America; The University of North Carolina at Chapel Hill, United States of America

## Abstract

The differentiation of cells into distinct cell types, each of which is heritable for many generations, underlies many biological phenomena. White and opaque cells of the fungal pathogen *Candida albicans* are two such heritable cell types, each thought to be adapted to unique niches within their human host. To systematically investigate their differences, we performed strand-specific, massively-parallel sequencing of RNA from *C. albicans* white and opaque cells. With these data we first annotated the *C. albicans* transcriptome, finding hundreds of novel differentially-expressed transcripts. Using the new annotation, we compared differences in transcript abundance between the two cell types with the genomic regions bound by a master regulator of the white-opaque switch (Wor1). We found that the revised transcriptional landscape considerably alters our understanding of the circuit governing differentiation. In particular, we can now resolve the poor concordance between binding of a master regulator and the differential expression of adjacent genes, a discrepancy observed in several other studies of cell differentiation. More than one third of the Wor1-bound differentially-expressed transcripts were previously unannotated, which explains the formerly puzzling presence of Wor1 at these positions along the genome. Many of these newly identified Wor1-regulated genes are non-coding and transcribed antisense to coding transcripts. We also find that 5′ and 3′ UTRs of mRNAs in the circuit are unusually long and that 5′ UTRs often differ in length between cell-types, suggesting UTRs encode important regulatory information and that use of alternative promoters is widespread. Further analysis revealed that the revised Wor1 circuit bears several striking similarities to the Oct4 circuit that specifies the pluripotency of mammalian embryonic stem cells. Additional characteristics shared with the Oct4 circuit suggest a set of general hallmarks characteristic of heritable differentiation states in eukaryotes.

## Author Summary

The differentiation of cells into distinct cell-types, each of which is “remembered” for many generations, underlies the development of both healthy and cancerous tissues. Such differentiation, however, is not restricted to multi-cellular organisms: “white” and “opaque” cells of the unicellular fungal pathogen *Candida albicans* are two heritable cell-types, each thought to be adapted to unique niches within their human host. Here we examine the differences between these two cell-types by sequencing their RNA contents and subsequently reconstructing and comparing their gene expression profiles. We know that the transcription factor Wor1 plays a central role in mediating these expression differences. As with many other transcriptional regulators, however, a major unresolved issue is the apparent discordance between the genomic locations to which Wor1 binds and whether neighboring genes are differentially expressed. Here we resolve this discordance, showing that hundreds of Wor1 binding sites, previously without apparent function, actually flank differentially-expressed genes that were undiscovered, or not measured accurately, before. Additionally, we find that transcripts regulated by Wor1 have many unusual properties, several of which we also observe for transcripts regulated during the development of mammalian embryonic stem cells, suggesting they may be general hallmarks of cell differentiation.

## Introduction

**Figure 1:**
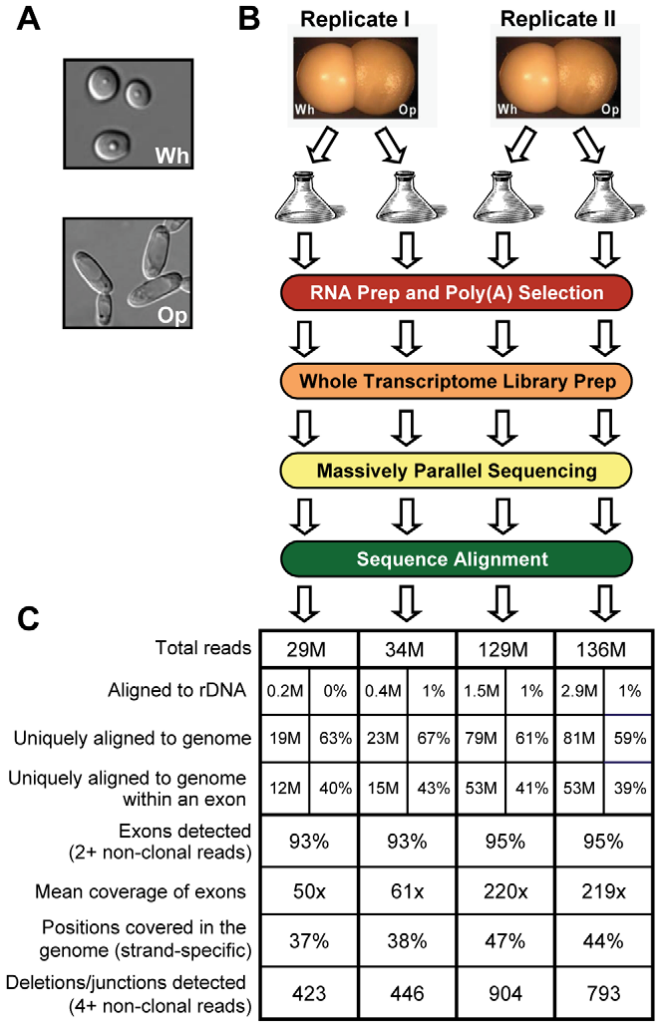
RNA sequencing of white and opaque cells. (A) White and opaque cells have distinct morphologies. (B) Summary of experimental design. (C) Summary statistics for alignments of RNA sequence reads. Read counts listed are expressed in millions (left column) or as a percentage of the total reads processed (right column) for each sample.

How differentiated cell types are epigenetically maintained through repeated cell division is a topic of intensive study [1], [2], both for its role in basic developmental processes [3] and its relevance to the advancement of human stem cell therapeutics [4]. However, as a basic model of differentiation, stem cell systems have several drawbacks, such as the vast number of distinct cell types, the difficulty of isolating large homogeneous cell populations, and the challenge of genetic manipulation. A much simpler example of epigenetic inheritance of differentiated cell states is found in *Candida albicans*, the most prevalent human fungal pathogen. This eukaryote forms two distinctive types of cells, white and opaque, that differ strikingly in their appearance [5] (Figure 1A and 1B), competency to mate [6], and the human tissues to which they are likely best suited [7]–[11]. Each cell type is heritably maintained through many cell divisions, with switching back and forth between the two cell types occurring stochastically, only once every 10^4^ generations. The low rate of switching makes it easy to obtain large populations of homogeneous cells of each type. Furthermore, it is relatively straightforward to manipulate the genes of *C. albicans*, which has allowed dissection of both the regulation underlying the switch and the functions of downstream genes that are ultimately responsible for conferring the specific attributes of each cell type [12]–[16] (for reviews, see [17], [18]).

A master regulator of the white-opaque switch, White Opaque Regulator 1 (Wor1), forms interlocking feedback loops with two other transcription regulators (Czf1 and Wor2). The three regulators are up-regulated in opaque cells compared to white cells and together are responsible for the establishment and maintenance of the opaque cell type [13]. The white state is maintained by the transcription regulator Efg1, which is down-regulated in opaque cells [13], [19]. The expression of more than 400 genes was previously found to differ between the two cell types [20], [21], but subsequent genome-wide chromatin immunoprecipitation (ChIP-Chip) experiments indicated that Wor1 directly bound only 58 of these genes [13]. Much of this discordance may be due to indirect regulation; indeed, Wor1 itself controls a large number of transcriptional regulators that may direct the differential expression of additional genes. However, it was much more difficult to explain the observation that only 30% of all Wor1-bound regions flank at least one differentially expressed transcript. Are the other Wor1 binding sites simply non-functional? Do they act only on more distal transcripts and/or only in response to certain environmental cues? Does Wor1 also play a non-regulatory role, helping to maintain chromosome structure via these binding sites? Although we investigate this issue specifically in *C. albicans*, we note that discordance between binding (determined by ChIP) and regulation (based on RNA analysis) has frequently been observed in the circuits of a broad range of organisms [22]–[26].

To better resolve the relationship between the binding of a master regulator of differentiation and differential expression of its direct targets between cell types, we performed massively-parallel strand-specific sequencing of RNA from white and opaque cells. Applying several novel algorithms to the resulting dataset and merging these results with the existing ORF-based gene annotation, we first annotated the *C. albicans* transcriptome. This revealed that thousands of transcripts overlap another transcript on the opposite strand, demonstrating widespread presence of anti-sense transcription in this yeast, as in the model yeast *Saccharomyces cerevisiae* [27], [28]. With the new annotation we found that the abundance of 1,306 transcripts differed between white and opaque cell types, a 3-fold increase over the number identified previously by microarray. We next revisited the poor correspondence between Wor1 binding and differential expression and found a remarkable improvement in concordance. Thus, a large fraction of the Wor1 bound regions previously lacking proximity to a differentially expressed gene, and therefore also lacking obvious function, can now be assigned the function of regulating previously invisible or inaccurately-measured transcripts.

Our analysis of the Wor1 circuit revealed several unusual properties. For example, the targets of Wor1 have abnormally long upstream intergenic regions and un-translated regions (UTRs). We show here that many of these long UTRs are cell-type-specific (that is, the transcript length is differentially regulated) and thus may function to bring additional layers of regulation to the differentiation circuit. A meta-analysis of the Oct4 circuit [29]–[31], which governs the pluripotency and differentiation of mouse embryonic stem cells, reveals many of these same “unusual” properties. These surprising similarities across vast evolutionary distances, combined with many other shared features, suggest that several hallmarks of cell differentiation circuits exist broadly across eukaryotes.

## Results

### The white and opaque transcriptomes

To characterize the transcriptomes of white and opaque cells, we sequenced the poly(A) fraction of RNA extracted from replicate white and opaque cell cultures (Materials and Methods and Figure 1B), expecting to find messenger RNAs, polyadenylated non-coding RNAs, and abundant non-polyadenylated transcripts that persist through the purification steps. Importantly, the sequencing libraries were prepared using an approach that preserves the genomic strand from which the sequenced RNA fragments were originally transcribed (see Materials and Methods and Figure S1) [32]. Our sequencing runs yielded 29–136 million 50-base sequence reads per sample, which were subsequently aligned to a filter database (containing, e.g., rDNA sequences) and then to the *Candida albicans* genome (build Ca21) and a database of previously annotated splice junctions (Materials and Methods and Figure S2). An overview of the results is depicted in Figure 1C. The majority of reads from each sample (60–68%) was successfully aligned, allowing detection of 93–95% of previously annotated exons with mean 50–200x sequence coverage (i.e., the number of reads aligned across a genomic position). 37–47% of positions were covered by an alignment in the strand-specific genome, and 423–904 deletions, which represent both splice junctions and deletion polymorphisms relative to the haploid reference genome, were detected (Mitrovich et al. [33], in preparation). On the whole, we have obtained more than sufficient sequence depth from these samples to build the first transcript annotation for *C. albicans*.

### *Candida albicans* transcript annotation

**Figure 2:**
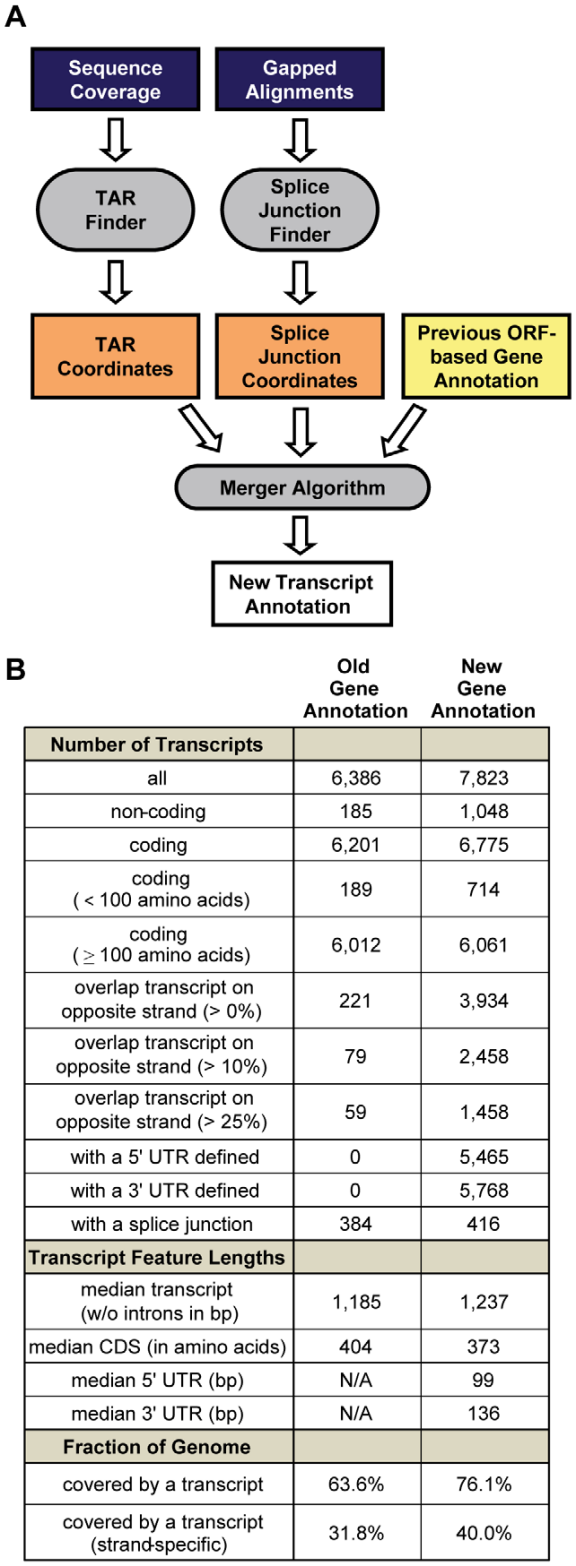
Defining a new transcript annotation for *C. albicans*. (A) Summary of computational workflow. (B) Summary statistics comparing the old ORF-based and new RNA-Seq-based transcript annotations.

Our RNA-Seq dataset allows us the first opportunity to define a true transcript annotation for *C. albicans*, which until now has had a gene annotation based primarily on computationally-predicted open reading frame (ORF) sequence boundaries and generally not informed by experimental data. We first developed a general computational approach (Figure 2A) that can define a new transcript annotation by combining an existing annotation (in this case the ORF-based annotation) with evidence found in RNA sequence data for un-translated regions (UTRs) and entirely novel transcripts. This effort included the development of new methods for the *de novo* identification of splice junctions and transcriptionally active regions (TARs), which are based on gapped read alignments and clusters of sequence coverage, respectively (Materials and Methods, Figure S3, and Mitrovich et al. [33], in preparation). We applied these methods to a single dataset produced by combining the reads from all four RNA sequence libraries, reasoning that (1) combining the datasets at this stage would be more powerful and straightforward than combining four separate annotations further downstream, and (2) the different datasets were sufficiently similar to one another. This is supported by the high reproducibility of biological replicates (r = 0.95−0.99; Figure S5) and the observation that most exons, when expressed in both cell types, appear to extend to roughly the same boundaries.

Rather than providing a completely *de novo* gene annotation (as for *S. cerevisiae* in Yassour et al. [34], for example), we sought to leverage the existing ORF-based annotation to provide an updated annotation in which existing transcripts, if expressed, were augmented with 5′ and 3′ UTRs, and new, isolated clusters of expression (i.e., those not overlapping an annotated exon on the same strand) were added to the annotation as novel TARs (nTARs). Thus, we devised a method to merge the splice junction and TAR-finding output with the existing ORF-based annotation (Materials and Methods and Figure S4) and applied it to our datasets, resulting in the new *C. albicans* transcript annotation (Tables S1, S2, S5; summarized in Figure 2B).

The new transcript annotation contains 23% more transcripts (N = 7,823) covering 13% more of the genome (76.1% versus 63.6%) than the old annotation. We estimate that roughly 1,048 of these transcripts are non-coding because they do not contain a canonical ORF that is at least 120 nucleotides long (i.e., encoding a peptide at least 40 amino acids long), which increases the number of non-coding RNAs (ncRNAs) annotated in *C. albicans* by nearly 500%. However, there are also a large number of new coding transcripts (i.e., transcripts that contain putative ORFs encoding peptides 40 or more amino acids long), leading to an estimated 9% increase in the number of coding transcripts. Many of these ORFs may have been missed in previous annotations due to their short length (91% are shorter than 100 amino acids) and, in some cases, due to lack of conservation in other species. It is likely that some of the ORFs defined here by our arbitrary length cutoff are not translated into protein. On the whole though, the number of putative ORFs at least 40 amino acids long found in novel transcripts (N = 561) is significantly higher than expected by chance (median N = 453; P-value <0.0001 by simulation; Materials and Methods), suggesting that many are translated into protein. As detailed in the next section, at least 18 of these short, novel ORFs are likely to serve an important function in opaque cells.

In the new transcript annotation 5′ and 3′ UTRs of median length 99 and 136 bases were defined for 5,465 and 5,768 transcripts, respectively. These estimates are longer than estimates of 5′ and 3′ UTR length based on tiling arrays (68 and 91 in David et al. [35]), but closely resemble those based on RNA-Seq data (111 and 142 in Yassour et al. [34]) for the related model yeast, *Saccharomyces cerevisiae*. Finally, 50% of transcripts in the new annotation overlapped transcripts from the opposite strand by at least 1 bp and 31% did so across more than 10% of their length, indicating that, as in other eukaryotes [27], [28], [36], there is widespread antisense transcription in *C. albicans*. This observation underscores the importance of sequencing RNA in a strand-specific manner. Overall, the new transcript annotation described here represents a dramatic revision from previous annotations that microarrays were designed to assess. Using this new annotation we revisited the differences in gene expression between white and opaque cells.

### Transcripts differentially expressed between white and opaque cell types

**Figure 3:**
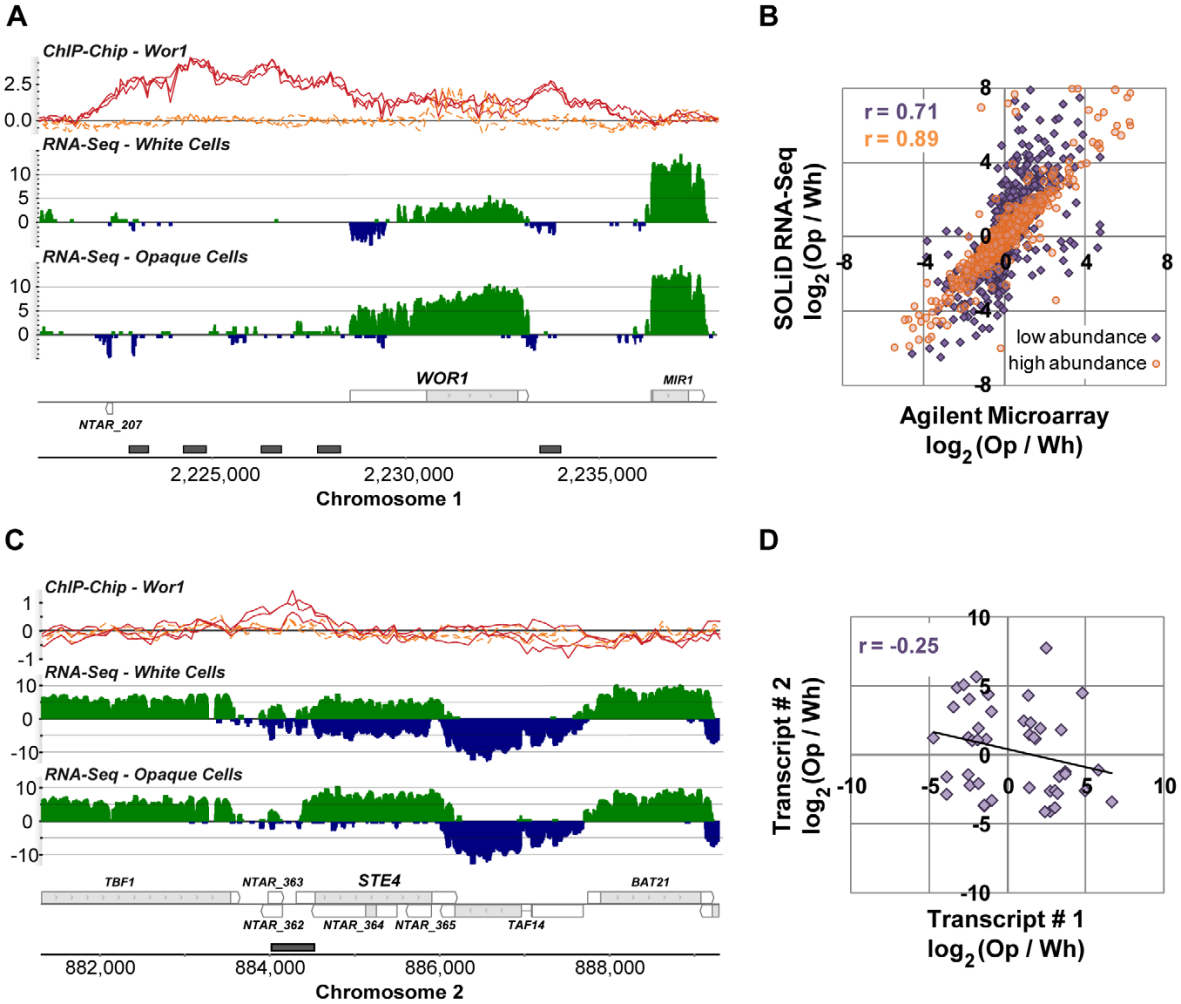
Transcripts differentially expressed between white and opaque cell types. (A) Expression and Wor1 enrichment at the *WOR1* locus as visualized in the MochiView Genome Browser [68]. In this and all other genomic plots presented here, Wor1 ChIP-Chip data are plotted in the top row (red-curves are from biological replicates of the Wor1 IP in opaque cells and orange curves are from IPs in *wor1*Δ Δ strains; normalized log_2_ IP DNA/Input DNA enrichment values are plotted), followed by RNA-Seq data for white and opaque cells (colored green on the plus and blue on the minus strand; values plotted are log_2_ sequence coverage), followed by transcript definitions in our new annotation (gray regions are coding and white are un-translated), and finally regions determined to be Wor1-bound by the peak finding algorithm (gray boxes). For interested readers, a MochiView database export of all the data presented in this work is provided at http://johnsonlab.ucsf.edu/mochi_files/Tuch_et_al_2010_PLoS_Genetics.cvw. (B) Comparison of RNA-Seq and microarray measurements of differential transcript expression (for previously annotated transcripts only). Transcripts are colored by their mean abundance across samples as measured by RNA-Seq: purple indicates mean RPKM ≤30 and orange indicates mean RPKM >30. (C) The expression of *STE4* is anti-correlated with the expression of its antisense transcript. (D) The expression of sense-antisense transcript pairs is only modestly anti-correlated (ρ = −0.25; P-value = 0.05).

We determined which of the 7,823 newly defined transcripts were differentially expressed between white and opaque cell types by employing a likelihood ratio test [37]. We required a 2-fold or greater change in expression and false discovery rate (FDR) of 10^−4^ or less, which resulted in a set of 1,306 differentially-expressed transcripts (Table S3). As expected, we find strong (50-fold) up-regulated expression of *WOR1*, the gene that encodes a master regulator of white-opaque switching (Figure 3A). As predicted by a previous study [14], *WOR1* has an unusually long 5′ UTR (1,978 bp, compared to the genome-wide median length of 99 bp). Unexpectedly, the lower *WOR1* expression in white cells is associated with increased expression on the strand opposite this long UTR, suggesting an alternative internal antisense promoter is active and may be repressing *WOR1* expression in white cells.

To confirm the quality of these data we compared them directly to data generated using microarrays that are commonly used to study gene expression in *C. albicans*. We hybridized the same samples used for RNA sequencing (Materials and Methods) and examined the fold-change measurements produced by each technology for all previously annotated transcripts (Figure 3B). We found a strong overall correlation (r = 0.79), which, as noted in other comparisons of RNA-Seq and microarray data, is stronger for high abundance transcripts (r = 0.89) than it is for low abundance transcripts (r = 0.71), which are generally more accurately measured by RNA-Seq [32], [37], [38].

The 1,306 differentially expressed transcripts found here represent a 3-fold increase in the number observed by microarray [21], which is partly attributable to the fact that 37% of these transcripts are novel (N = 488) and thus were not probed on previous microarrays. Novel transcripts are unexpectedly frequent amongst the set of white-opaque differentially-expressed transcripts (N = 488 versus 218 expected; χ^2^ P-value = 10^−89^), a provocative observation we can not yet entirely explain, but which suggests an important role for non-coding transcripts and short proteins in the white-opaque circuit. In any case, this observation emphasizes the importance of “hypothesis-free” approaches to measuring gene expression. The remaining differentially-expressed transcripts, not recognized as such by microarray (N = 376), may be explained by the documented, improved sensitivity and dynamic range of RNA-Seq [38], [39]; indeed, these transcripts not discovered by microarray have 2-fold lower average abundance than those that were, as estimated by RPKM (reads per kb of transcript per million uniquely aligned reads).

**Figure 4:**
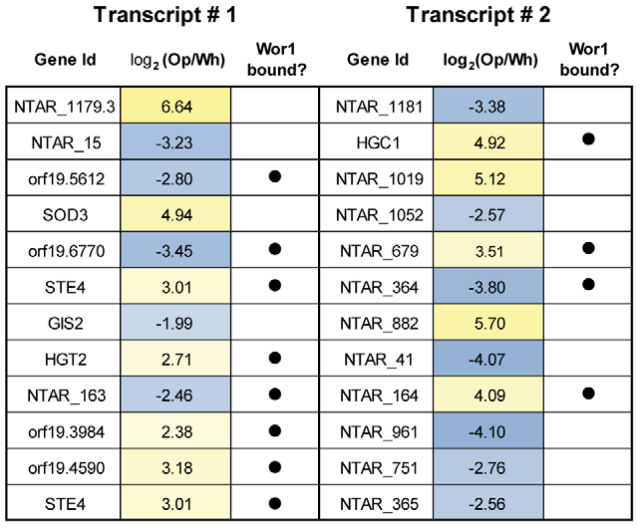
A selection of sense-antisense gene pairs with the most strongly anti-correlated expression. Each row lists a sense-antisense transcript pair, the differential expression in opaque versus white cells for each transcript in the pair, and whether or not each transcript is Wor1 bound.

We were especially interested in the 488 novel differentially expressed transcripts, which fall into three major classes: (1) antisense transcripts, (2) isolated transcripts that encode proteins, and (3) isolated non-coding transcripts. We discuss these three classes in turn. We found 213 novel transcripts that overlap another transcript on the opposite strand across at least one third of their length. *NTAR_364* is a particularly informative example of a differentially expressed novel transcript that overlaps another transcript on the opposite strand (Figure 3C). The gene opposite *NTAR_364* is *STE4*, which encodes the β subunit of the heterotrimeric G protein complex required for mating [40], [41]. Mating is a process specific to opaque cells [6], and accordingly, *NTAR_364*'s 14-fold down-regulation is inverse to *STE4*'s 8-fold up-regulation in opaque cells. The anti-correlated expression of these two overlapping transcripts strongly suggests a mechanism in which NTAR_364's expression acts to repress expression of STE4. There is ample precedent for this type of regulation in eukaryotes and bacteria [42]–[45]. To determine the prevalence of such mechanisms in *C. albicans*, we examined the expression profiles of all 759 such sense-antisense transcript pairs, filtering down to the subset of 44 pairs in which both transcripts are significantly changed and at least one transcript is coding (Figure 3D). Our expectation was that we would observe strong anti-correlated differential expression across all such pairs if these mechanisms are prevalent and a lack of correlation if they are not. Instead, we found a modest and significant anti-correlation (r = −0.25; P-value  = 0.05; Figure 3D). Sense–antisense pairs in which one member is differentially-expressed are 2-fold more likely, than expected by chance, to have the second member differentially-expressed in the opposite direction (17% versus 8%; χ^2^ P-value  = 10^−4^). These results suggest that some, but not all, anti-sense transcripts act to repress the steady-state abundance of their sense counterpart. Despite the lack of perfect anti-correlation, there are several transcript pairs that, like the *STE4*-*NTAR_364* pair mentioned, are considerably differentially-expressed in opposite directions (Figure 4), which strongly suggests a regulatory function for the novel antisense transcripts involved.

**Figure 5:**
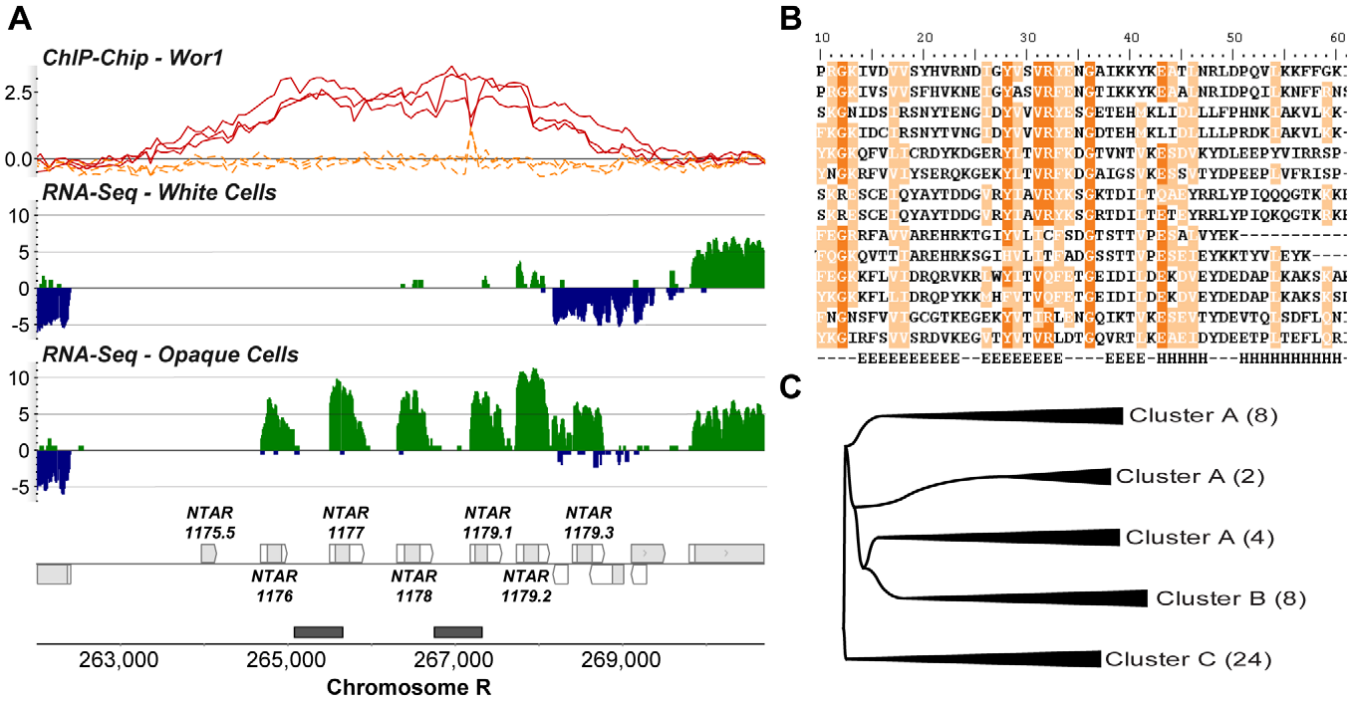
Three clusters of novel *Candida*-specific ORFs are strongly up-regulated in opaque cells. (A) Expression and Wor1 binding at cluster A, the *NTAR_1176* locus (others shown in Figure S6A and S6B), containing 7 novel ORFs on the positive strand, 6 of which are expressed only in opaque cells. (B) Partial multiple sequence alignment of all members of cluster A (see Figure S6C for alignment of all 46 homologs) in *C. albicans* and *C. dubliniensis*. The predicted secondary structure is noted in the final row (E  =  β sheet and H  =  α helix) [47]. (C) Compressed neighbor-joining phylogeny of all 46 *NTAR_1176* homologs found in *C. albicans* and *C. dubliniensis* (see Figure S6D for full tree).

The second major class of novel, differentially-expressed transcripts contains those that are isolated in the genome and code for protein. In total, we identified 224 novel differentially expressed transcripts that do not overlap a transcript on the opposite strand. Sixty-nine of these transcripts encode a putative protein at least 40 amino acids long. Amongst these is a group that clusters into three genomic locations and encodes a large family of novel, short ORFs (Figure 5A, Figure S6A and S6B). Eighteen of the 24 ORFs in this family are encoded by transcripts that are opaque-specific, including *NTAR_1179.2*, which with 287-fold higher abundance in opaque cells is the third most differentially-expressed transcript genome-wide. Using a combination of BLAST and PSI-BLAST against fungal genomes and eukaryotic protein sequence databases, we identified 46 members of this family (see sequence alignments in Figure 5B and Figure S6C), 24 from *C. albicans* and 22 from its closest known relative, *Candida dubliniensis*. Homologs could not be identified in any other species, further underscoring the potential importance of these genes to opaque-cell differentiation, since these two yeast species are the only two known to switch between distinct white and opaque forms [46]. The neighbor-joining phylogeny inferred for these ORFs (Figure 5C and Figure S6D) indicates that most were present and similarly clustered in the common ancestor of *C. albicans* and *C. dubliniensis*. Computational predictions of secondary structure [47] indicated there are likely three β sheets followed by two α helices in these proteins (Figure 5B) and the structure prediction server I-TASSER [48] found a putative bacterial hemolysin (PDB ID: 3HP7) to be the closest structural analog.

Finally, 155 of the isolated, differentially-expressed transcripts do not appear to code for protein. At this time it is difficult to assess their functions in a purely computational manner; thus, their roles in the white-opaque switch await experimental characterization.

In all three classes of novel transcripts we observe examples in which the master regulator Wor1 is bound adjacent to or overlapping the differentially expressed transcripts (Figure 3C and Figure 5A), suggesting that these novel antisense and isolated transcripts are directly regulated by Wor1 binding. Thus, they may form a key, but heretofore unknown, part of the circuit.

### The new transcript annotation illuminates the Wor1 circuit

**Figure 6:**
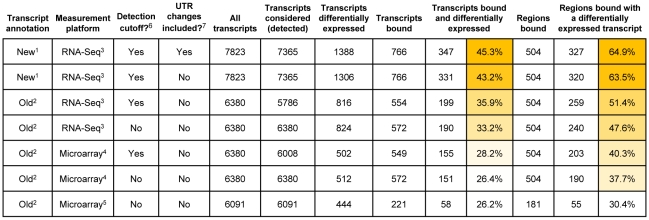
Association of Wor1 binding with white-versus-opaque differential expression when different transcript annotations and measurement platforms are employed. An RNA-Seq-based annotation with RNA-Seq-based differential expression measurements (top row) provides the strongest concordance between differential expression and Wor1 binding. Footnotes: **1** The transcript annotation derived from RNA-Seq data in this work. **2** The previous ORF-based gene annotation from Candida Genome Database (CGD). **3** Differential expression measurements from RNA-Seq data reported in this work. **4** Differential expression measurements from hybridization to custom Agilent 8x15k microarrays reported in this work. **5** Differential expression measurements from hybridization to spotted cDNA microarrays reported previously [21]. **6** Indicates whether or not a gene expression detection threshold was employed to filter putatively dubious transcripts from the annotation prior to computing the association between binding and differential expression. **7** Indicates whether or not the genes detected as having UTR length changes between the cell types are considered “differentially-expressed.” Note that such genes may or may not be differentially expressed in the traditional sense (i.e., when considering the entire transcript or just the coding region of the transcript).

To assess the concordance between Wor1 binding and differential expression of nearby transcripts more globally we compared the previous ORF-based and our new RNA-Seq-based gene annotations to regions identified as Wor1-bound in chromatin immunoprecipitation-on-tiling microarray (ChIP-Chip) experiments [13]. We first associated Wor1-bound regions with adjacent genes using both the new and the old annotations (Figure S7), and then evaluated both the frequency with which Wor1 binding flanked at least one differentially expressed gene and the frequency with which Wor1-bound genes were differentially expressed (Figure 6). We also compared measurements of differential expression from three different platforms: (a) hybridization to spotted PCR-product microarrays (reported previously by Tsong et al. [21]), (b) hybridization to custom-designed Agilent 8x15k microarrays (reported here), and (c) strand-specific RNA-Seq (also reported here). The pairing of the new transcript annotation with the RNA-Seq measurements of differential expression (Figure 6, first row) clearly yields the strongest concordance between Wor1 binding and differential expression: 65% of Wor1-bound regions are associated with at least one differentially expressed transcript. This represents a greater than 2-fold improvement in concordance over a previously published association [13], in which only 30% of bound regions were observed to flank at least one differentially expressed transcript (Figure 6, last row). In this previous association, differential expression of transcripts was measured by spotted PCR-product arrays designed to assay only transcripts in the old annotation. The concordance between binding and differential expression improves incrementally with the use of better microarray platforms (38–40%; Figure 6, rows 5–6) and with RNA-Seq-based expression measurements computed using the old transcript annotation (48–51%; Figure 6, rows 3–4). However, by far the best concordance is found when RNA-Seq-based expression measurements are computed using the new transcript annotation. Thus, the dramatically improved association of master regulator binding and cell type-specific expression observed here is attributable to both the novel transcripts and the improved expression measurements provided by RNA-Seq.

### Unusual properties of the Wor1 circuit

**Figure 7:**
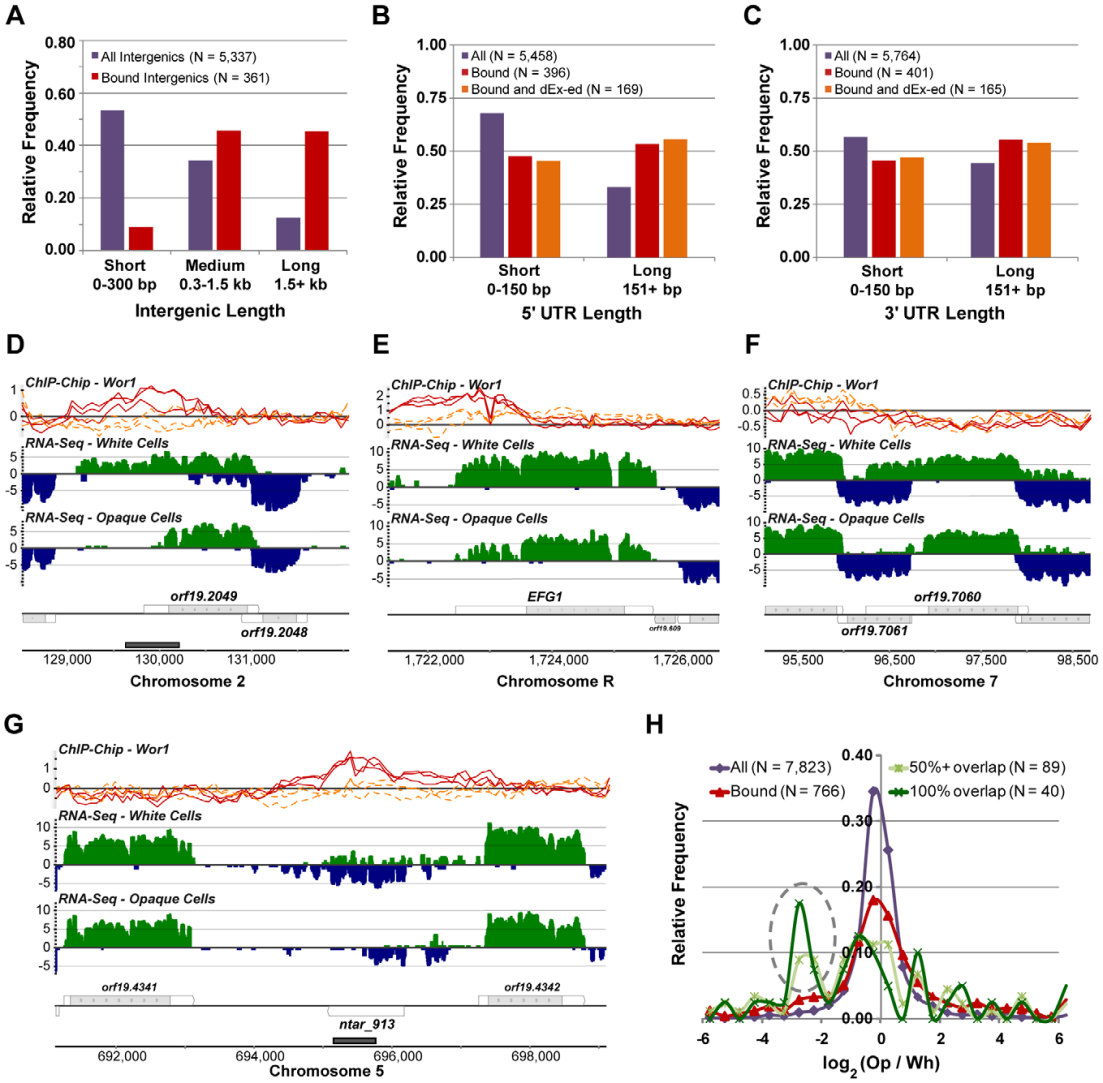
Properties of transcripts in the Wor1 circuit. (A) The distribution of lengths for all intergenic regions and Wor1-bound intergenic regions. The distribution of lengths for the (B) 5′ UTRs and (C) 3′ UTRs of all transcripts, transcripts associated with Wor1 binding, and transcripts that are associated with Wor1 binding and differentially expressed (“dEx-ed”) between white and opaque cells. Expression and Wor1 binding at three genes with apparent changes in UTR length between the cell types: (D) *ORF19.2049*, (E) *EFG1*, and (F) *ORF19.7060*. (G) Expression and Wor1 binding at the *NTAR_913* locus, an example of a gene for which down-regulation in opaque cells is correlated with overlapping binding of Wor1. (H) The distribution of differential expression (opaque versus white fold-changes) for all transcripts, transcripts associated with Wor1 binding, and transcripts that are directly overlapped at least 50% or 100% by Wor1 binding. The gray dashed oval highlights an enriched subset of transcripts for which overlapping Wor1 binding is correlated with down-regulation in opaque cells.

The fact that the *WOR1* gene has a 2 kb long 5′ UTR and about 6 kb of Wor1-bound intergenic DNA upstream of it (Figure 3A) suggests that this master regulator of white-opaque switching is under complex regulation. We next examined whether other transcripts in the circuit have similar properties. It was previously noted that Wor1-bound intergenic regions are, on average, 5-fold longer than typical intergenic regions (median 3,390 bp for Wor1-bound genes versus 623 bp genome-wide) [13]. However, given the substantial changes we have made to the gene annotation, it was unclear whether this length bias would remain; in particular, it seemed plausible that some of the unusually long “intergenic” regions may actually contain, and thus be due to, previously unannotated long UTRs. We find that while genome-wide intergenic length is, on average, more than 2-fold shorter in the new annotation (new median length  = 262 bp), the intergenic regions bound by Wor1 are still, on average, 5-fold longer than expected by chance (new median length  = 1346 bp; Mann-Whitney P-value  = 10^−80^; Figure 7A). Unexpectedly, we also found that 5′ UTRs of Wor1-bound genes are 58% longer than expected (median 157 bp in the circuit versus 99 bp genome-wide; Mann-Whitney P-value  = 10^−20^; Figure 7B) and 3′ UTRs in the circuit are 22% longer than expected (median 166 bp in the circuit versus 136 bp genome-wide; Mann-Whitney P-value  = 10^−6^; Figure 7C).

The unusually long UTRs found in the Wor1 circuit and the apparent change in UTR length at *WOR1* (Figure 3A) motivated us to look more generally into changes in promoter usage and transcriptional termination between cell types, as reflected in changes in 5′ and 3′ UTR length, respectively. We devised a simple method to isolate putative cases of UTR length change, reasoning that a change in UTR length for a given transcript could be detected as a change in the *apparent* expression of the UTR that is significantly less than or greater than what was measured for the transcript's coding region. We required a minimum 2-fold difference in fold-change between UTR and coding region and a χ^2^ P-value less than 10^−5^ (Materials and Methods). Using these criteria, we identified 145 transcripts with at least one UTR apparently changing length between white and opaque cells (Table S4). Visual inspection revealed that not all these cases are straightforward to interpret; however, many are, and these provide several examples for further study (Figure 7D–7F). Most of the cases identified here are changes in 5′ UTRs (N = 111; 77%), which likely reflects an emphasis on the usage of alternative promoters as a means of differentiating the two cell types. One of the transcripts, *EFG1*, is a regulator of white-opaque switching and was previously shown to exhibit different 5′ UTR lengths in white and opaque cells [49]. *EFG1* and 26 other transcripts with significant 5′ UTR changes are also associated with Wor1 binding nearby their genomic loci (observed frequency  = 24%; expected  = 10%; χ^2^ P-value  = 10^−8^). For several of these transcripts, such as *ORF19.2049* (Figure 7D) and *EFG1* (Figure 7E), the UTR is shorter in opaque cells and Wor1 is bound in opaque cells between the apparent white- and opaque-preferred transcription start sites, suggesting a direct regulatory mechanism. Other examples, such as *PPS1* (not shown) and *ORF19.7060* (Figure 7F), are probably not directly related to Wor1 binding, but may instead involve mechanisms related to the transcription of antisense genes.

Comparing Wor1 binding to gene expression revealed an additional feature of Wor1-controlled transcripts: direct binding of Wor1 within a transcribed region (rather than upstream of it) is associated with strong down-regulation of the bound transcript in opaque cells. The non-coding transcript NTAR_913 provides a clear example of this phenomenon (Figure 7G). Genome-wide, we found 89 cases in which a transcript overlaps a Wor1-bound region by more than 50%, and the expression of such transcripts is frequently white-specific (Figure 7H). This observation suggests the prominence of an underappreciated mode of gene regulation in which a transcription regulator may repress transcription via direct binding to the transcribed region. Given the unusual characteristics of the *WOR1* locus and Wor1's target genes, we next examined whether other examples of heritable cell differentiation circuits exhibited similar features.

### Unusual properties of the Oct4 circuit governing mammalian differentiation

**Figure 8:**
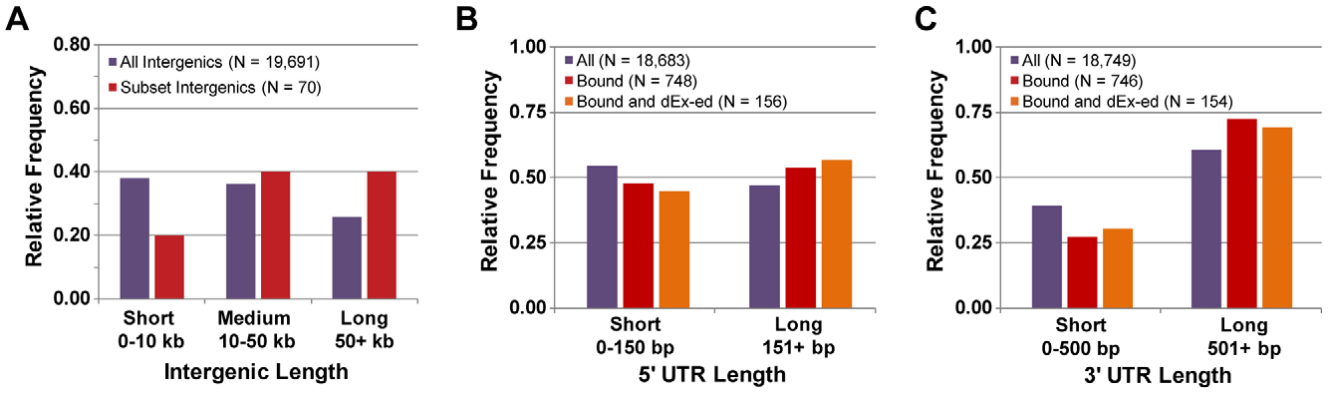
Properties of transcripts in the Oct4 circuit. (A) The distribution of lengths for all intergenic regions and intergenic regions that are associated with Oct4 binding. The distribution of lengths for the (B) 5′ UTRs and (C) 3′ UTRs of all transcripts, transcripts associated with Oct4 binding, and transcripts that are associated with Oct4 binding and differentially expressed (“dEx-ed”) during differentiation.

One of the most studied transcription circuits is that of Oct4, which governs the differentiation and pluripotency of mammalian embryonic stem (ES) cells [1], [50]. Oct4 is a master regulator of mammalian cell types in the same sense that Wor1 is a master regulator of *Candida* cell types: Oct4 expression is required to maintain the pluripotent ES cell type [51], and Oct4's over-expression in other cell types, along with additional factors, returns them to the ES cell state [2], [52]. Although much is known about this circuit, we could not find any previous reports on the general properties of the circuit (e.g., relative UTR length of Oct4-bound genes). To determine if the unusual properties of the Wor1 circuit in *Candida* are shared with the Oct4 circuit, we performed a meta-analysis of publicly-available data, including ChIP-Seq-based Oct4 binding data [30], [31] and microarray-based profiles of gene expression during stem cell differentiation [29] (Materials and Methods). We discovered that the Oct4 circuit of mice does indeed share “unusual” characteristics with the Wor1 circuit of *Candida*. Intergenic regions bound by Oct4 are 33% longer than expected by chance (median 23 kb in the circuit versus 17 kb genome-wide; Mann-Whitney P-value  = 10^−3^) and are 2-fold longer than expected if they also flank a transcript that is differentially expressed during differentiation (median 34 kb in the differentially-expressed circuit; Mann-Whitney P-value  = 10^−4^; Figure 8A). 5′ UTRs and 3′ UTRs are also longer than expected (161 and 1048 bp in the circuit versus 137 and 727 bp genome-wide; Mann-Whitney P-values  = 10^−5^ and 10^−12^, respectively; Figure 8B and 8C), but the relative magnitude of length bias for 5′ versus 3′ UTRs (+18% and +44%, respectively) is flipped relative to that observed in the Wor1 circuit (+58% and +22%, respectively). Unfortunately, the appropriate data are not yet available to determine whether UTR lengths are frequently changing between cell types in the Oct4 circuit of mice as they are in the Wor1 circuit of *Candida*.

## Discussion

By sequencing the transcriptomes of white and opaque cells (Figure 1) and applying a novel computational approach (Figure 2A), we have provided the first transcript annotation for *C. albicans* (Figure 2B), the most prevalent human fungal pathogen. This new view of the *C. albicans* transcriptional landscape includes over a thousand newly discovered transcripts, some of which are transcribed antisense to previously annotated genes, but many of which are entirely isolated from other genes. A subset of these transcripts codes for proteins, some of which are specific to *Candida* species and may function in host-pathogen interactions. Overall, the new view of gene expression in *C. albicans* is reminiscent of that provided by recent sequencing of the transcriptome of another yeast species, *S. cerevisiae* [28], [34], [38], but with two important differences. First, we have captured a more faithful depiction of the transcriptome by using a method that measures expression across entire genes in a strand-specific fashion. Second, relative to the model organism *S. cerevisiae*, the transcriptome of *C. albicans* was poorly characterized prior to RNA sequencing. Our analysis dramatically expands the view of transcription in this yeast, resulting in annotations for hundreds of new coding and non-coding transcripts and thousands of UTRs.

The revised annotation and expression data allowed us to examine, at unprecedented resolution, the differences between two cell types. White and opaque cells are specified by one of the largest known transcriptional circuits in *C. albicans*; as discussed in the introduction, each cell type is heritable for many generations and switching between them is epigenetic. Our principle findings are summarized as follows:

1. Between white and opaque cells, hundreds of previously unidentified transcripts are differentially-expressed. Most are apparently non-coding, but a substantial fraction appears to code for short, previously unrecognized proteins. On the whole, we found 3-fold more differentially expressed transcripts than were previously identified by microarray analysis. Part of this difference can be attributed to the identification of new transcripts and part to the greater sensitivity and dynamic range offered by the RNA-Seq approach used here [32], [37]–[39], [53].
2. Among the new coding transcripts, perhaps most interesting are 24 that encode a family of short proteins (Figure 5). The transcripts encoding these proteins are nearly absent in white cells (median RPKM  = 0.1), but abundant in opaque cells (median RPKM  = 10.5). The presence of family members only in the two species known to have distinct white and opaque forms suggests a recent *de novo* origin, followed by an expansion via gene duplication. Although we do not yet know the function of these short proteins, it seems likely, based on their narrow distribution in pathogenic fungal species, that they are intimately linked to the adaptation of opaque cells to their niche within the human host.
3. Many of the non-coding, differentially-expressed RNAs are antisense to mRNAs. In some cases the transcripts in these sense-antisense pairs display anti-correlated differential expression between cell types (Figure 4), which likely indicates regulation via transcriptional interference mechanisms. For example, *STE4*, which encodes a signaling protein required for the opaque-specific developmental process of mating [40], [41], is strongly up-regulated in opaque cells coincident with the strong down-regulation of its antisense transcript (Figure 3C). This example is reminiscent of the antisense regulation observed in *S. cerevisiae* of *IME4* [45], which controls initiation of meiosis, the complementary developmental fate. Other non-coding RNAs are suggestive of different types of regulatory mechanisms (see below).
4. The integration of our RNA-Seq data with genome-wide ChIP data provides a new understanding of the relationship between the binding of a master transcription regulator (Wor1) and the differentiated transcriptomes it specifies. We found substantial (>100%) improvement of the concordance between Wor1 binding and the differential expression of nearby transcripts compared with our earlier analysis (Figure 6). This new information greatly clarifies the function of hundreds of Wor1-occupied sites in the genome that were previously unexplained. It would not be surprising if many of the binding sites proposed to be “non-functional” in other transcription circuits [22]–[26] turn out to regulate transcripts that were not previously observed or whose expression differences were not accurately measured. This analysis also revealed that many of the direct targets of the master regulator Wor1 are non-coding RNAs, suggesting an important role for regulatory RNAs in specifying the two cell types.
5. The Wor1-regulated mRNAs show unusually large 5′ and 3′ UTRs (Figure 7B and 7C), suggesting that post-transcriptional regulation is a prevalent, although previously overlooked, component of the regulatory circuit.
6. Many mRNAs exhibit different 5′ UTRs in white and opaque cells (Figure 7D–7F), indicating the widespread use of alternative promoters in specifying the two cell types.

In addition to the conclusions listed above, a comparison of the RNA-seq data from *C. albicans* to those determined in other species reveals some important differences and similarities. With the new strand-specific data presented here we were able to systematically examine changes in the expression of sense and antisense transcripts. The high frequency of antisense transcripts combined with the weak anti-correlated expression of transcripts in sense-antisense pairs (Figure 3D) suggests that while transcriptional interference mechanisms likely control transcription rates in some cases, antisense transcription may also play a different role in this yeast, perhaps acting post-transcriptionally via RNAi mechanisms Genome-wide anti-correlated expression of sense-antisense pairs was previously observed in *S. cerevisiae* [27], but in that study the anti-correlation across all sense-antisense pairs was stronger than what we observed here. It is possible that the difference between species is related to the loss of mechanisms for post-transcriptional control by antisense transcripts in *S. cerevisiae*, but not in *C. albicans* [54]. Thus, whereas *C. albicans* may use antisense transcripts for a mix of transcriptional and post-transcriptional regulation, antisense transcription in *S. cerevisiae* may function primarily to regulate sense transcripts through transcriptional interference.

Finally, we note several striking mechanistic similarities between the Wor1 circuit that governs white-opaque switching in yeast and the Oct4 circuit that controls the pluripotency and differentiation of mammalian embryonic stem cells. In both systems, differentiation is controlled by a series of master transcription regulators arranged in interlocking feedback loops, the differentiation process requires long periods of time relative to the cell division time, and the differentiated states are “remembered” through many cell generations [1], [17], [18], [50]. In each system, hundreds of binding sites for the master regulator were thought to be “non-functional” [25], though, as we have shown here for the yeast system, many of these instead are likely to impart cell-type specific expression to previously unannotated transcripts. In addition, amongst the direct targets of the master regulators is an abundance of genes that encode transcription regulators themselves [13], [29], [55] and genes with unusually long upstream intergenic regions (compare Figure 7A to Figure 8A) and abnormally long UTRs (compare Figure 7B and 7C to Figure 8B and 8C). It seems likely that the latter two characteristics reflect a large number of regulatory inputs to genes of these circuits. The expanded upstream regions may also allow the formation of more complex tertiary chromatin structures involved in gene regulation [56], [57]. Regardless of their function, they are clearly identifiable landmarks of both circuits. We have also shown here that many of the long UTRs are regulated, in the sense that they are longer in one cell type and shorter in the other. Finally, it appears as though non-coding RNAs are an important component of both circuits [31]. Taken together, these findings suggest an unexpected level of sophistication is required to maintain distinct cell types through many cell divisions—whether in a relatively simple fungal system with only two cell types, or in a complex mammalian developmental system involving numerous differentiated tissues.

## Materials and Methods

### RNA sample preparation

White cells of mating type **a**/**a** were selected by growth of *C. albicans* strain QMY23 [58], a derivative of the sequenced strain SC5314, on sorbose medium [59]. Opaque cell lines were then isolated following spontaneous cell-type switching. Liquid cultures of white or opaque cells (two samples of each, referred to throughout the manuscript as white and opaque replicate #1 and white and opaque replicate #2) were grown at 23°C in SC medium [60] supplemented with 100 mg/l uridine to an OD_600_ of 1 (log phase growth). Samples (5 ml) were collected by centrifugation (5 min, 2000 *g*, 4°C), and pellets frozen in liquid nitrogen. Total RNA was extracted from frozen pellets as described [61]. For each sample, poly(A) RNA was isolated from 50 µg of total RNA by two rounds of purification using a Poly(A)Purist MAG kit (Ambion).

### Whole transcriptome (WT) library preparation

To construct libraries suitable for SOLiD System sequencing (Figure S1), each poly(A)-selected RNA sample (150–300 ng) was fragmented in a 10 µl volume by incubation with 1 unit of RNase III and 1X reaction buffer (Ambion) for 10 minutes at 37°C. Fragmented RNA was then immediately diluted to 100 µl and purified using a RiboMinus Concentration Module (Invitrogen) following manufacturer's protocol, with the following modifications: sample was initially mixed with 100 µl Binding Buffer and 250 µl ethanol, column was washed only once with 500 µl Wash Buffer, and purified sample was eluted in 20 µl water. RNA fragmentation was confirmed and sample quantified using an Agilent 2100 Bioanalyzer, with an RNA 6000 Pico Chip, following manufacturer's protocol. 50 ng fragmented RNA was dried by vacuum centrifugation at low heat, then suspended in 3 µl water. An amplified cDNA library was constructed using components from the SOLiD Small RNA Expression Kit (Ambion). Hybridization and ligation of Adaptor Mix A to the fragmented RNA and reverse transcription were carried out according to manufacturer's protocol, but with 18 h ligations and no RNase H treatment. cDNA was brought up to 100 µl and purified using a Qiagen MiniElute PCR Purification Kit, following manufacturer's protocol. Half of the eluted cDNA was mixed with an equal volume of loading dye (95% formamide, 0.5 mM EDTA, 0.025% each bromophenol blue and xylene cyanol FF), heated to 95°C for 3 min, then cooled immediately on ice. Sample was run on a 7 cm denaturing 7M urea/1X TBE/6% polyacrylamide gel at 180V for 17 min, then stained with SYBR Gold Nucleic Acid Gel Stain (Invitrogen). DNA was visualized by UV-illumination, and material between 100–200 nt excised by scalpel. The excised region was further cut into 4 vertical strips (such that each represented the same DNA size distribution). Amplification was performed directly on gel strips again using components from the SOLiD Small RNA Expression Kit (Ambion). Two 100 µl PCR reactions were performed, each with one gel strip, 1X PCR Buffer, 0.2 mM dNTP mix, 2 µl AmpliTaq DNA Polymerase, and 2 µl SOLiD PCR Primer Sets 1, 2, 3 or 4 (for white and opaque sample replicates #1 and white and opaque sample replicates #2, respectively). Reactions conditions were 95°C (5 min); 16 cycles of 95°C (30 sec), 62°C (30 sec), and 72°C (30 sec); 72°C (7 min). The two amplification reactions were pooled and purified using a PureLink PCR Micro Kit (Invitrogen) following manufacturer's protocol, but combining two sequential elutions. To ensure appropriate size distributions (>75% of product >140 bp), products were assayed using a Bioanalyzer DNA 1000 chip; yields ranged from 360–1140 ng.

### Emulsion PCR and sequencing of WT libraries

Templated beads were generated for sequencing using standard manufacturers' protocols. Beads from the first pair of white and opaque libraries (“Replicate #1”) were deposited onto a full slide with 8 other barcoded libraries not presented here. Beads from the second pair of white and opaque libraries (“Replicate #2”) were deposited onto two quadrants of a slide each. Massively parallel ligation sequencing was carried out to 50 bases using Life Technologies SOLiD System V3 and following the manufacturer's instructions.

### Hybridization of cDNA to microarrays

For microarray analysis, we used aliquots of the same total RNA samples used to generate the WT libraries (replicate #2; discussed above). Aminoallyl-labeled cDNAs were synthesized using 5 µg of total RNA in 50 µl reverse transcription reactions with 250U SuperScript III Reverse Transcriptase (Invitrogen), as described previously [58]. The cDNA samples were dried in a speed-vac to ≤9 µl total. Samples were then brought to 9 µl with water and supplemented with 1 µl of fresh 1M Na Bicarbonate, pH 9.0. Cy3 and Cy5 dyes were prepared by re-suspending Amersham mono-reactive dye packs (Cat. #PA23001 and PA25001) in 10 µl DMSO, and 1.25 µl of either Cy3 or Cy5 were added to each sample. Labeling reactions were incubated for one hour at room temperature in darkness. Dye-coupled cDNA samples were purified by adding 800 µl of Zymo DNA binding buffer (Zymo Research) to each sample and loading onto Zymo-25 columns. The remainder of the purification was performed as per the manufacturer's directions, and the samples were eluted with 40 µl of water. For each competitive hybridization, 0.2 µg each of Cy3 and Cy5 labeled cDNA were combined in 25 µl final volume of water, incubated at 95°C for 3 min, cooled to room temperature, mixed with 25 µl of Agilent 2x GE hybridization buffer (HI -RPM), and loaded onto individual “blocks” (40 µl each) on Agilent 8x15k custom gene expression microarrays. Hybridization was carried out at 65°C for 16 hours and the arrays were washed with Agilent wash buffers as per the manufacturer's recommendations.

### Alignment of transcriptome reads

Whole transcriptome reads were aligned to a modified version of the Assembly 21 release of the *Candida albicans* genome [62]. As this is a haploid assembly, known single nucleotide variation between alleles from the most recent diploid assembly (Assembly 19, [63]) was mapped to Assembly 21, and the genome sequence was modified to reflect these ambiguous positions, allowing expressed sequences from either allele to be aligned equivalently. Alignment was performed with Life Technologies' SOLiD Whole Transcriptome Pipeline [32], [64]. This software is open-source and freely available (http://solidsoftwaretools.com/gf/project/transcriptome/). An overview of the alignment strategy is presented in Figure S2. In all the analyses of gene expression presented here, only reads that were both uniquely and fully aligned were considered. A “uniquely and fully” aligned read is defined as a read with a max-scoring alignment to the genome (1) scoring at least 31 (alignment score is calculated with a match score of +1 and a mismatch score of −2), (2) scoring at least 9 higher than any of the other alignments of that read to the genome, and (3) at least 40 bp long. All sequence data have been deposited at the MIAME compliant Gene Expression Omnibus (GEO) database at the National Center for Biotechnology Information (http://www.ncbi.nlm.nih.gov/geo) and are accessible through accession number GSE21291.

### Finding splice junctions

Known and novel splice junctions were identified by looking for sets of read sequences whose alignments share a gap (specifically, a deletion relative to the reference) with the same genomic start and end coordinates. We determined empirically that by requiring at least 5 such reads, and considering only deletions of at least 50 nucleotides, we captured, and thus validated, 85% of the 421 known junctions, while also predicting 158 novel junctions or deletions. False positives were filtered from this set by requiring matches to splice motifs and by removing deletions caused by obvious artifacts (e.g., cleavage and polyadenylation junctions), yielding 45 new introns in total. The details of this method are provided elsewhere in Mitrovich et al. (In preparation) [33].

### Finding putative transcriptionally active regions (pTARs)

A TAR is a region of the strand-specific genome exhibiting a cluster of sequence coverage, most often representing the presence of an exon. We employed a sliding window approach to identify such clusters on each strand of the *C. albicans* genome. The approach is described in depth in the manual for Life Technologies' Novel Transcribed Region (NTR) finder (http://solidsoftwaretools.com/gf/download/docmanfileversion/138/693/NTR_Finder_Manual_v1.1.pdf). Briefly, a window of specified size is scanned base-by-base across the genome, average sequence coverage is calculated within each window, and windows with average coverage greater than a specified cutoff are marked. A set of contiguous marked regions in the genome is then joined and trimmed from each end to better fit the coverage profile, forming a putative TAR (pTAR). We used the NTR finder to perform TAR-finding on the combined dataset of all four sequence libraries presented in this work. TAR-finding was performed with many different parameter sets (i.e., different values chosen for the size of the window and the minimum average coverage required for the marking of a region) and it was determined that a window size of 125 and minimum average coverage of 20 were optimal for reproducing the previously annotated TARs (aTARs), with the expectation that the pTARs would be slightly larger than the aTARs because the existing annotations were ORF-based only and thus did not include UTR definitions (Figure S3). Other parameters were kept fixed: min-score = 25, trimming-fraction = 0.01, min-overlap = 0.9. The existing transcript annotation (Ca21), which is primarily based on putative ORF sequences, was downloaded from the Candida Genome Database (http://www.candidagenome.org/) and the exons defined therein were used as our aTARs.

In merging the pTARs with aTARs to define a new transcript annotation, we found that in addition to this optimal pTAR set (pTAR_opt_set, with parameters window-size = 125, min-window-coverage = 20, min-score = 25, trimming-fraction = 0.01, and min-overlap = 0.9), a more fragmented pTAR set produced from a smaller window size (pTAR_frag_set, with parameters window-size = 10, min-window-coverage = 20, min-score = 25, trimming-fraction = 0.01, and min-overlap = 0.9) was also helpful (see below).

We also experimented with Hidden Markov Model (HMM) approaches to finding pTARs (not shown), but found that the models we trained did not perform better than the simpler sliding window approach taken here. In fact, they tended to perform much worse, which may simply reflect that we did not find the best way of modeling the segmentation problem.

### Merging pTARs and novel splice junctions with the existing ORF-based transcript annotation (aTARs) to form the new transcript annotation

Rather than providing a completely *de novo* transcript annotation [34], we sought to leverage the existing annotation to provide an updated transcript annotation in which existing ORF-encoding regions, if expressed, were augmented with 5′ and 3′ UTRs and isolated TARs (i.e., those not overlapping an aTAR on the same strand) were added to the transcript annotation as novel TARs (nTARs). Thus, we employed a set of rules that merged the pTAR_opt_set with the aTARs in the previous transcript annotation (Ca21, from the Candida Genome Database [65]) to form a new set of transcript annotations. The rules are most concisely described diagrammatically in Figure S4. For transcripts found to contain one or more splice junctions, the internal exon coordinates defined by reads spanning those splice junctions are used in place of those defined by the pTARs (i.e., splice junction-derived coordinates override these purely coverage-based coordinates). The more fragmented pTAR_frag_set was used to define transcript boundaries in cases where two or more aTARs were overlapped by a single pTAR (scenario ‘f’ in Figure S4), which typically happens when transcripts are positioned very close to one another on the same strand. In such cases, if a pTAR was found in the more fragmented set that overlapped the edge of one aTAR without also overlapping the edge of the other aTAR, this pTAR was used to define the UTR of the overlapping aTAR in the new annotation.

### Simulation of expected number of ORFs found in nTARs

We performed 10,000 rounds of simulation to determine whether the 561 nTARs containing an ORF of length 40 amino acids or longer was more than expected by chance. In each round, 1,443 regions with the same size distribution as the 1,443 nTARs were chosen randomly in a strand-specific fashion from regions of the genome not covered by ORFs in the previous annotation (i.e., the Ca21 ORF-based annotation). The median number of ORFs found per round was 453. 561 or more ORFs were not found in any round of the simulation (P-value <0.0001).

### Differential expression between cell types from RNA–Seq data

For each transcript model (in either the new or old annotation), reads that uniquely aligned to the genome within its exons or across its splice junctions were counted. One pseudo-count was added to this sum and the resulting modified raw transcript count was converted to a normalized measurement of abundance by normalizing for transcript length and total number of uniquely aligned reads in the sample (i.e., RPKM; reads aligned per kb of transcript per million uniquely aligned reads) [39], [66]. The fold-change of each transcript between cell types was then computed by dividing its mean RPKM across opaque cell replicates by its mean RPKM across white cell replicates. We employed a recently proposed likelihood ratio test combined with a fold-change cutoff to define sets of differentially expressed transcripts [37]. Specifically, a false discovery rate (FDR) less than or equal to 10^−4^ and an absolute fold-change greater than or equal to 2 defined a set of 1306 differentially expressed transcripts using the new transcript annotation and a set of 824 using the old annotation. RPKM, fold-change estimates, P-values and FDRs for each transcript can be found in Table S3.

### Differential expression between cell types from microarray data

Microarray data were normalized and differentially expressed transcripts were identified using limma v2.16.5 [67] in R v2.8.1. Background correction was performed with the “normexp” method and an offset value of 50. Normalization was then performed within arrays using the “loess” method and between arrays using the “quantile” method. Finally, differential expression of transcripts between white and opaque cells was determined on our dye-swapped replicate arrays using the “lmFit” and “eBayes” methods, which produced fold-change estimates and Benjamini-Hochberg multiple test-corrected P-values for each probe on the array. For each transcript, only the expression value given by the probe with the highest average expression value (i.e., AveExpr value) was used in downstream analysis. As with the analysis of the RNA-Seq data, we applied an adjusted P-value cutoff of 10^−4^ and required an absolute fold-change greater than or equal to 2. This defined a set of 512 differentially expressed transcripts.

### Defining Wor1-bound regions from ChIP–Chip data

Wor1-bound regions were identified as peaks of binding enrichment in the Wor1 ChIP-Chip data using the “Extract peaks from Data Set(s)” utility of MochiView v1.311 [68]. The algorithm is described in detail in the MochiView manual. Briefly, a smoothing function is applied to the log_2_ enrichment values of the Wor1 ChIP-Chip tiling arrays followed by the application of an algorithm to detect local regions of maximal enrichment (i.e. binding peaks), which are assigned a P-value using permutation testing. Note that this algorithm is not based on deconvolution of binding events using shearing profiles – in the case of the Wor1 ChIP-chip data, the binding peaks are atypically broad and varied, and thus tend to confound deconvolution-based algorithms. Peak extraction was applied independently to the normalized ChIP-Chip data derived from antibodies targeting the N- and C-terminus of Wor1 [13]. Peak-finding significance thresholds were kept at their default values (P≤0.001 in the Wor1 ChIPs of wild-type cells and P>0.05 in the Wor1 ChIPs of *wor1*ΔΔ controls), though the amount of sampling was increased 10-fold from default to improve significance estimates. The minimum value for peak inclusion/consideration was set to 0.25. All other settings were kept at their default values. It was subsequently determined that the union of Wor1-bound regions defined independently from the N- and C-terminal datasets gave the best concordance with microarray-based and RNA-seq-based gene expression measurements of differential expression. Thus, the 504 Wor1-bound regions used throughout this work result from taking the union of Wor1-bound regions generated from the N- and C-terminal ChIP-Chip datasets.

### Associating transcription factor (TF) binding with putatively regulated overlapping and flanking transcripts

For the purposes of comparing Wor1 binding to differential expression, Wor1-bound regions were associated with nearby divergently transcribed transcripts as depicted in Figure S7.

### Analysis of transcript features in the Wor1 circuit

For the purposes of calculating the distribution of intergenic lengths “in the Wor1 circuit” a slightly different approach was taken to associate Wor1-bound regions with nearby transcripts than described above. In this case, Wor1-bound regions that fall within intergenic regions were associated with all divergent transcripts within 1 kb and intergenic regions that associated with one or more such transcripts were determined to be “in the Wor1 circuit”. This approach avoids the problem of length correction required under the null model that binding sites are distributed randomly throughout the genome (i.e., that longer intergenic regions are inherently more likely to have random binding). Similarly, to avoid length bias when determining the distribution of 5′ and 3′ UTR lengths “in the Wor1 circuit”, we only considered Wor1-bound regions that resided in the intergenic space immediately upstream of the transcript, thereby avoiding the possibility that random binding to the longer UTRs themselves would drive artificial UTR length discrepancies.

### Differential UTR lengths between cell types

Putative cases of UTR length change between cell types were isolated by comparing changes in UTR expression to changes in coding sequence (CDS) expression between the cell types. We first calculated differential expression (in white versus opaque cells) independently for the 5′ un-translated, coding, and 3′ un-translated regions of each coding transcript. The number of reads aligned within each region of a transcript was counted in the merged set of alignments from each cell type (i.e., the two biological replicates for each cell type were combined) and a single pseudocount was added. The counts for the opaque cell type, whose dataset had 4% more uniquely aligned reads overall, were normalized by the ratio of uniquely aligned reads in the datasets of the two cell types (i.e., they were multiplied by a constant factor of 0.96). Fold-changes were calculated for each transcript region by dividing the normalized count in opaque by the count in white cells. We then scanned for UTRs whose expression changed more or less than their corresponding coding sequence, as determined by a χ^2^ test of independence comparing the observed, normalized UTR counts to the expected counts in the two cell types. The expected count for each CDS region in each cell type was calculated by redistributing the total reads counted across cell types for the corresponding UTR in a fashion proportional to the fold-change calculated for the CDS. To ensure accurate fold-change estimates for the CDS regions, only transcripts with a CDS that had at least 50 reads aligned in at least one cell type were considered. By also requiring a minimum 2-fold absolute difference in fold-change values for the UTR and CDS regions and a χ^2^ P-value less than 10^−5^, we identified 145 transcripts with putative UTR length changes (Table S4).

### Meta-analysis of transcript features in the Oct4 circuit

The analysis of transcript features in the Oct4 circuit was performed on publicly available data. Lists of Oct4-bound regions in mouse ES cells determined independently by Chen et al. [25] and Marson et al. [31] were downloaded from supplemental tables provided by these groups in their respective publications. The intersection of bound regions from these two sources was taken to define a high confidence set of Oct4-bound regions that was used for all further analysis. Gene expression measurements of differentiating mouse ES cells were downloaded from a supplemental table provided by Loh et al [29]. For the purposes of our analysis, we considered transcripts that were significantly (multiple test-corrected P-value ≤10^−4^) up- or down-regulated across the 18 profiling experiments (median fold-change of at least 1.5) to be differentially expressed between cell types. Mouse transcript annotations were downloaded from the UCSC Genome Browser (http://genome.ucsc.edu/) and are based on alignments of RefSeq transcripts to assembly mm8 of the mouse genome sequence [69]. The distribution of intergenic lengths “in the Oct4 circuit” was calculated as described above for the Wor1 circuit, except that in the mammalian circuit transcripts could be up to 10 kb away from an Oct4-bound region. We allow a longer distance here since intergenic regions are overall much longer in mouse and because regulation is generally expected to occur over longer distances. The distribution of 5′ and 3′ UTR lengths “in the Oct4 circuit” was calculated as described above for the Wor1 circuit.

## Supporting Information

Figure S1 RNA-Seq library workflow. The protocol used to prepare total RNA for SOLiD System sequencing is diagrammed here. This approach achieves strand-specificity by employing end-specific ligation of sequencing adapters to RNA, prior to the cDNA synthesis step. The P1 sequencing adapter is an RNA/DNA complex that contains a 6 bp 5′ single-strand DNA overhang allowing it to hybridize only to the 5′ end of an RNA fragment and, likewise, the P2 adapter will hybridize only to the 3′ end. The ligase used is engineered specifically to prefer the types of double-stranded substrates produced by these hybridizations, effectively making proper hybridization a prerequisite for efficient ligation. Thus, when cDNA is sequenced off the P1 adapter we can determine the genomic strand from which the RNA originated. Also, because RNA is fragmented prior to cDNA synthesis, the protocol is less biased with respect to the positional origin of fragments within transcripts.
(0.12 MB TIF)

Figure S2 Short read sequence alignment algorithm. RNA sequencing reads were analyzed using Life Technologies Whole Transcriptome software tools (http://solidsoftwaretools.com/gf/project/transcriptome/). Briefly, each 50 base read was broken into two pieces (consisting of bases 1–23 and 25–47; please note that for simplicity the figure depicts the simplified scenario in which each read is broken into two 25 bp halves) and each piece was mapped independently and contiguously to the *Candida albicans* genome (Ca21) and a database of annotated splice junction sequences. During this mapping phase we allowed up to three mismatches and removed reads that align to more than 100 locations. The mapping of each read piece was extended along the mapped genomic region using colors (i.e., di-base calls) from the rest of the read until a maximal score was reached (+1 for a match and −2 for a mismatch). In cases where the read pieces aligned to the same genomic location, the results from the two halves were merged. Reads that did not align “fully” (i.e., with an alignment score of at least 31 and an alignment length of at least 40) or uniquely after merger were passed through to the rescue phase. During rescue a read is re-aligned to the region extending 2 kb downstream of each position to which a read piece was contiguously mapped, this time allowing a single insertion in the read of up to 5 bases or deletion of up to 2 kb relative to the reference. This process is especially helpful for identifying novel splice junctions. Only reads that were aligned both uniquely and “fully” were subsequently used to generate counts for annotated exons, transcripts, and genes, as well as genomic coverage plots (WIG files) that were displayed in the MochiView Genome Browser [68].
(0.13 MB TIF)

Figure S3 Putative transcriptionally active region (pTAR) finder method and results. In the pTAR finding method a window of specified size is scanned base-by-base across the genome, average sequence coverage is calculated within each window, and windows with average coverage greater than a specified cutoff are marked. A set of contiguous marked regions in the genome is then joined and trimmed from each end to better fit the coverage profile, forming a putative TAR (pTAR). TAR finding was performed with many different parameter sets (i.e., different values chosen for the size of the window and the minimum average coverage required for the marking of a region) and the resulting pTAR sets were compared to annotated TARs (aTARs) from the previous ORF-based transcript annotation. (A) The fraction of aTARs that were “recovered” in the pTAR set for various window size (represented as series with different colors) and minimum average coverage (represented as the points within each series) values. “Recovered” aTARs must overlap a pTAR by at least 90%. (B) The average fraction of each aTAR that overlaps a pTAR across different pTAR sets. (C) The average fraction of each pTAR that overlaps an aTAR across different pTAR sets. Based on these plots, it was determined that a window size of 125 and minimum average coverage of 20 are optimal for reproducing the aTARs (panel A), with the expectation that the pTARs would be slightly larger than the aTARs (B,C) because the existing annotations were ORF-based only and therefore did not include UTR definitions.
(0.26 MB TIF)

Figure S4 Algorithm for merging putative pTARs (pTARs) with previously annotated TARs (aTARs). (A) The rules used to merge the pTARs and aTARs to form the new transcript annotation are depicted. For example, scenario ‘a’ is the “ideal” scenario in which a single RNA-Seq-based pTAR overlaps a single ORF-based aTAR, with the pTAR's coordinates extending past aTAR's coordinates on both the 5′ and 3′ ends, defining the un-translated regions (UTRs) of the transcript. The number of times each scenario was observed is listed in parentheses. For transcripts found to contain one or more splice junctions (see Methods), the internal exon coordinates defined by reads spanning those splice junctions are used in place of those defined by the pTARs (i.e., splice junction-derived coordinates override these purely coverage-based coordinates). Occasionally two or more aTARs were overlapped by a single pTAR (scenario ‘f’) in the optimal pTAR set (pTAR_opt_set; see Methods), which typically happens when transcripts are positioned very close to one another on the same strand thus leading to either only a small or no break in sequence coverage between the transcripts. In such cases, if a pTAR was found in the more fragmented set (pTAR_frag_set, defined with a smaller window-size parameter; see Methods) that overlapped the edge of one aTAR without also overlapping the edge of the other aTAR, this pTAR was used to define the UTR of the overlapping aTAR in the new annotation. After the rules depicted are applied, TARs assigned to scenario ‘b’ are merged with TARs in any scenario if they fall within 100 bp, which appears to help clean up fragmented long UTRs and yields a more conservative estimate of the total number of nTARs found. (B) An example genome plot illustrating how sequence coverage is used to call pTARs, which are in turn merged with aTARs from the old transcript annotation to form the new transcript annotation.
(0.85 MB TIF)

Figure S5 Reproducibility of fold-changes across biological replicates. The abundance of each transcript as estimated by RPKM (reads per kb of transcript per million uniquely aligned reads) from the sequencing of two independently grown (A) white and (B) opaque cell cultures.
(0.34 MB TIF)

Figure S6 Three clusters of novel Candida-specific ORFs are strongly up-regulated in opaque cells. (A) Expression and Wor1 binding at cluster B of NTAR_1176 homologs on chromosome R (“chrR”). (B) Expression and Wor1 binding at cluster C of NTAR_1176 homologs on chrR. (C) Multiple sequence alignment of all 46 NTAR_1176 homologs found by PSI-BLAST in *C. albicans* and *C. dubliniensis*. (D) Neighbor-joining tree of the 46 NTAR_1176 homologs. Clusters A, B, and C are shaded green, yellow, and blue, respectively.
(1.23 MB TIF)

Figure S7 Associating Wor1-bound regions with putatively regulated overlapping and nearby transcripts. Transcripts associated (shaded orange) and not associated (shaded white) with a flanking Wor1-bound region (shaded red) are indicated. Arrows indicate the inferred direction of transcription for each TAR.
(0.15 MB TIF)

Table S1 New transcript annotation.
(2.74 MB TXT)

Table S2 Hand-edited version of the new transcript annotation. Same as Table S1 except here we have manually modified the nTARs containing the short ORFs homologous to ntar_1176. This required splitting some nTARs and creating others that were not expressed under the conditions studied here. We also removed the UTRs of HIS1, which has been replaced in the studied strain with its ortholog from another species.
(2.74 MB TXT)

Table S3 Results of the differential expression (white versus opaque) analysis.
(0.87 MB TXT)

Table S4 Results of the differential UTR length (white versus opaque) analysis.
(1.04 MB TXT)

Table S5 Final new transcript annotation. Same as Table S2 except here we have unified the transcript naming scheme and manually modified the structure of one complex gene on the mitochondrial genome, CaalfMp08. This table was not used for any of the analyses mentioned in the paper; rather, the main purpose of this table is to allow interested readers to easily load a gene annotation into MochiView [68]. This is also the transcript annotation we intend to deliver to the Candida Genome Database (CGD) [65].
(2.73 MB TXT)
