# Supplementary figures and images for: The Transcriptomes of Two Heritable Cell Types Illuminate the Circuit Governing Their Differentiation
Source: PLoS Genet. 2010 Aug 19;6(8):e1001070. doi: 10.1371/journal.pgen.1001070 (PMC2924316; doi:10.1371/journal.pgen.1001070)

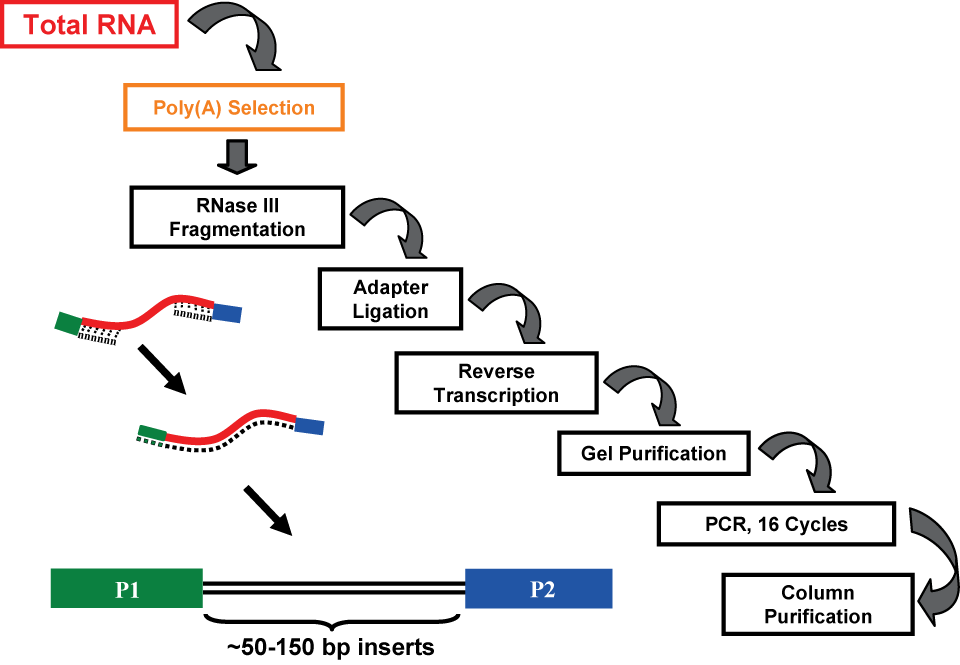

Supplement: Figure S1 — RNA-Seq library workflow. The protocol used to prepare total RNA for SOLiD System sequencing is diagrammed here. This approach achieves strand-specificity by employing end-specific ligation of sequencing adapters to RNA, prior to the cDNA synthesis step. The P1 sequencing adapter is an RNA/DNA complex that contains a 6 bp 5′ single-strand DNA overhang allowing it to hybridize only to the 5′ end of an RNA fragment and, likewise, the P2 adapter will hybridize only to the 3′ end. The ligase used is engineered specifically to prefer the types of double-stranded substrates produced by these hybridizations, effectively making proper hybridization a prerequisite for efficient ligation. Thus, when cDNA is sequenced off the P1 adapter we can determine the genomic strand from which the RNA originated. Also, because RNA is fragmented prior to cDNA synthesis, the protocol is less biased with respect to the positional origin of fragments within transcripts. (0.12 MB TIF) [file pgen.1001070.s001.tif]

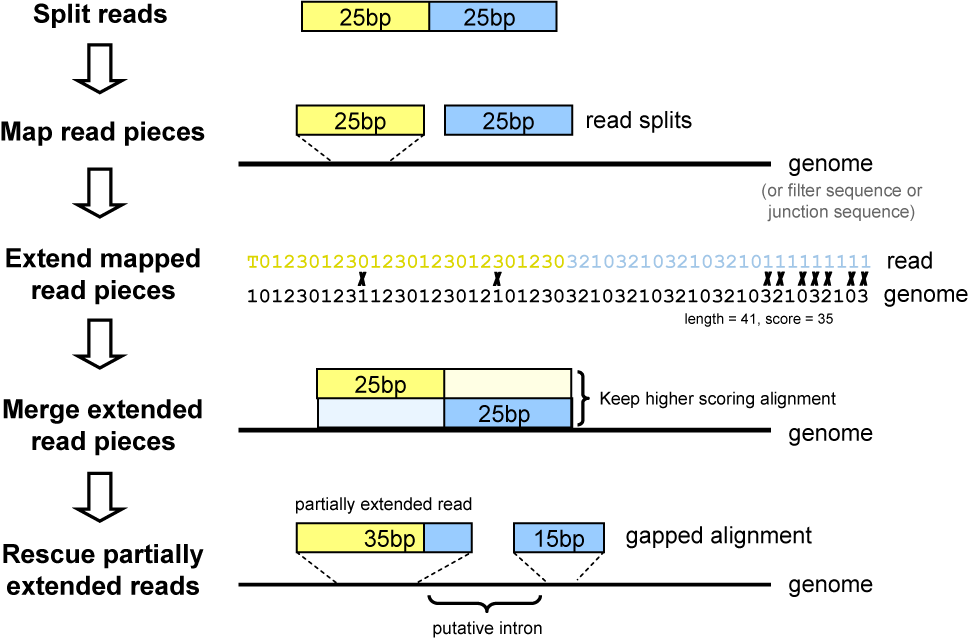

Supplement: Figure S2 — Short read sequence alignment algorithm. RNA sequencing reads were analyzed using Life Technologies Whole Transcriptome software tools (http://solidsoftwaretools.com/gf/project/transcriptome/). Briefly, each 50 base read was broken into two pieces (consisting of bases 1–23 and 25–47; please note that for simplicity the figure depicts the simplified scenario in which each read is broken into two 25 bp halves) and each piece was mapped independently and contiguously to the Candida albicans genome (Ca21) and a database of annotated splice junction sequences. During this mapping phase we allowed up to three mismatches and removed reads that align to more than 100 locations. The mapping of each read piece was extended along the mapped genomic region using colors (i.e., di-base calls) from the rest of the read until a maximal score was reached (+1 for a match and −2 for a mismatch). In cases where the read pieces aligned to the same genomic location, the results from the two halves were merged. Reads that did not align “fully” (i.e., with an alignment score of at least 31 and an alignment length of at least 40) or uniquely after merger were passed through to the rescue phase. During rescue a read is re-aligned to the region extending 2 kb downstream of each position to which a read piece was contiguously mapped, this time allowing a single insertion in the read of up to 5 bases or deletion of up to 2 kb relative to the reference. This process is especially helpful for identifying novel splice junctions. Only reads that were aligned both uniquely and “fully” were subsequently used to generate counts for annotated exons, transcripts, and genes, as well as genomic coverage plots (WIG files) that were displayed in the MochiView Genome Browser [68]. (0.13 MB TIF) [file pgen.1001070.s002.tif]

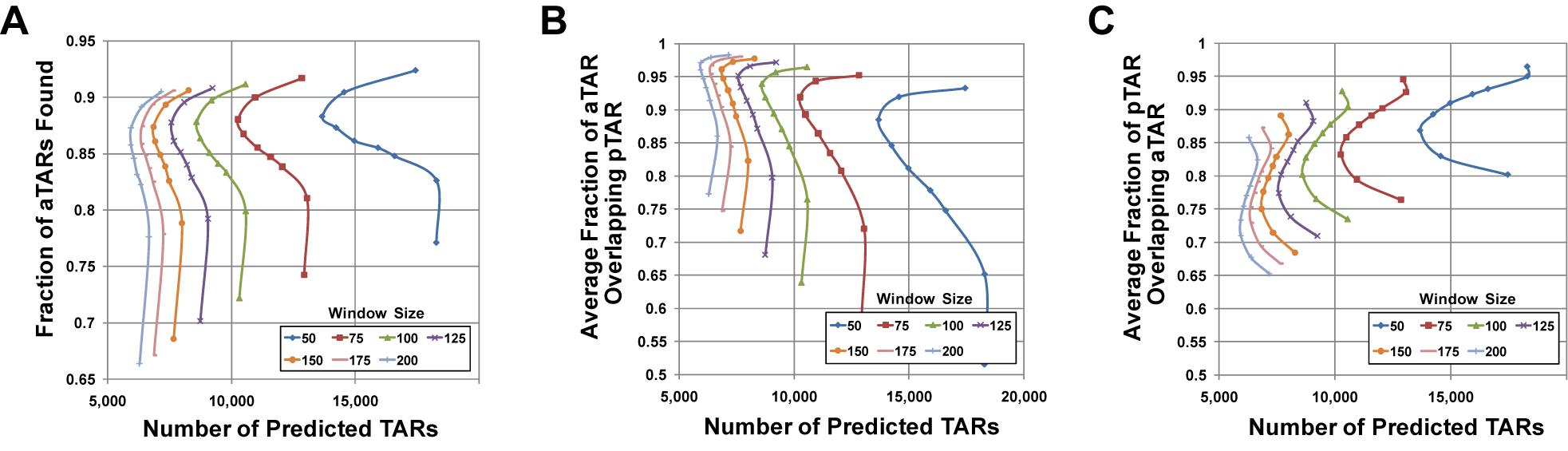

Supplement: Figure S3 — Putative transcriptionally active region (pTAR) finder method and results. In the pTAR finding method a window of specified size is scanned base-by-base across the genome, average sequence coverage is calculated within each window, and windows with average coverage greater than a specified cutoff are marked. A set of contiguous marked regions in the genome is then joined and trimmed from each end to better fit the coverage profile, forming a putative TAR (pTAR). TAR finding was performed with many different parameter sets (i.e., different values chosen for the size of the window and the minimum average coverage required for the marking of a region) and the resulting pTAR sets were compared to annotated TARs (aTARs) from the previous ORF-based transcript annotation. (A) The fraction of aTARs that were “recovered” in the pTAR set for various window size (represented as series with different colors) and minimum average coverage (represented as the points within each series) values. “Recovered” aTARs must overlap a pTAR by at least 90%. (B) The average fraction of each aTAR that overlaps a pTAR across different pTAR sets. (C) The average fraction of each pTAR that overlaps an aTAR across different pTAR sets. Based on these plots, it was determined that a window size of 125 and minimum average coverage of 20 are optimal for reproducing the aTARs (panel A), with the expectation that the pTARs would be slightly larger than the aTARs (B,C) because the existing annotations were ORF-based only and therefore did not include UTR definitions. (0.26 MB TIF) [file pgen.1001070.s003.tif]

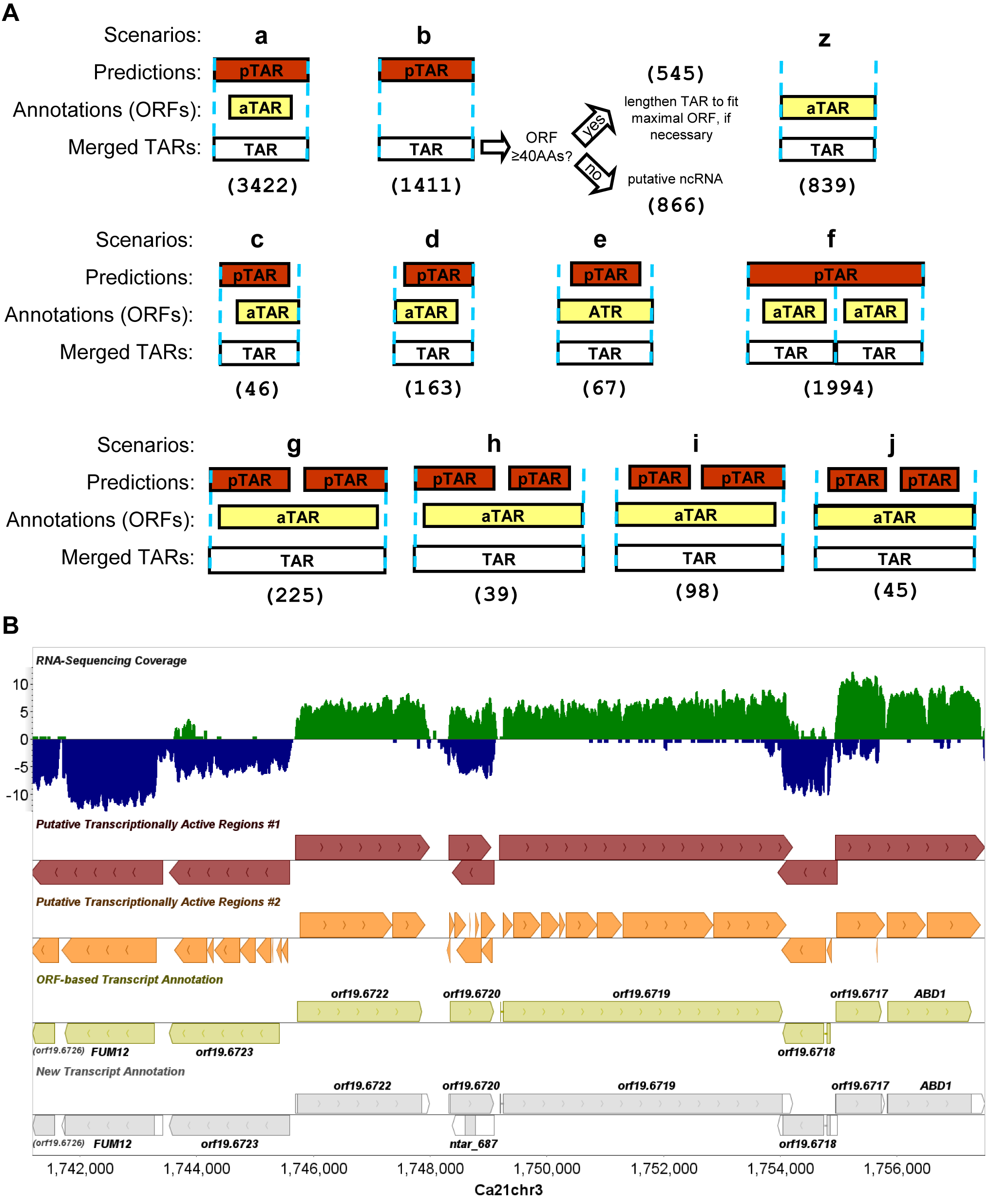

Supplement: Figure S4 — Algorithm for merging putative pTARs (pTARs) with previously annotated TARs (aTARs). (A) The rules used to merge the pTARs and aTARs to form the new transcript annotation are depicted. For example, scenario ‘a’ is the “ideal” scenario in which a single RNA-Seq-based pTAR overlaps a single ORF-based aTAR, with the pTAR's coordinates extending past aTAR's coordinates on both the 5′ and 3′ ends, defining the un-translated regions (UTRs) of the transcript. The number of times each scenario was observed is listed in parentheses. For transcripts found to contain one or more splice junctions (see Methods), the internal exon coordinates defined by reads spanning those splice junctions are used in place of those defined by the pTARs (i.e., splice junction-derived coordinates override these purely coverage-based coordinates). Occasionally two or more aTARs were overlapped by a single pTAR (scenario ‘f’) in the optimal pTAR set (pTAR_opt_set; see Methods), which typically happens when transcripts are positioned very close to one another on the same strand thus leading to either only a small or no break in sequence coverage between the transcripts. In such cases, if a pTAR was found in the more fragmented set (pTAR_frag_set, defined with a smaller window-size parameter; see Methods) that overlapped the edge of one aTAR without also overlapping the edge of the other aTAR, this pTAR was used to define the UTR of the overlapping aTAR in the new annotation. After the rules depicted are applied, TARs assigned to scenario ‘b’ are merged with TARs in any scenario if they fall within 100 bp, which appears to help clean up fragmented long UTRs and yields a more conservative estimate of the total number of nTARs found. (B) An example genome plot illustrating how sequence coverage is used to call pTARs, which are in turn merged with aTARs from the old transcript annotation to form the new transcript annotation. (0.85 MB TIF) [file pgen.1001070.s004.tif]

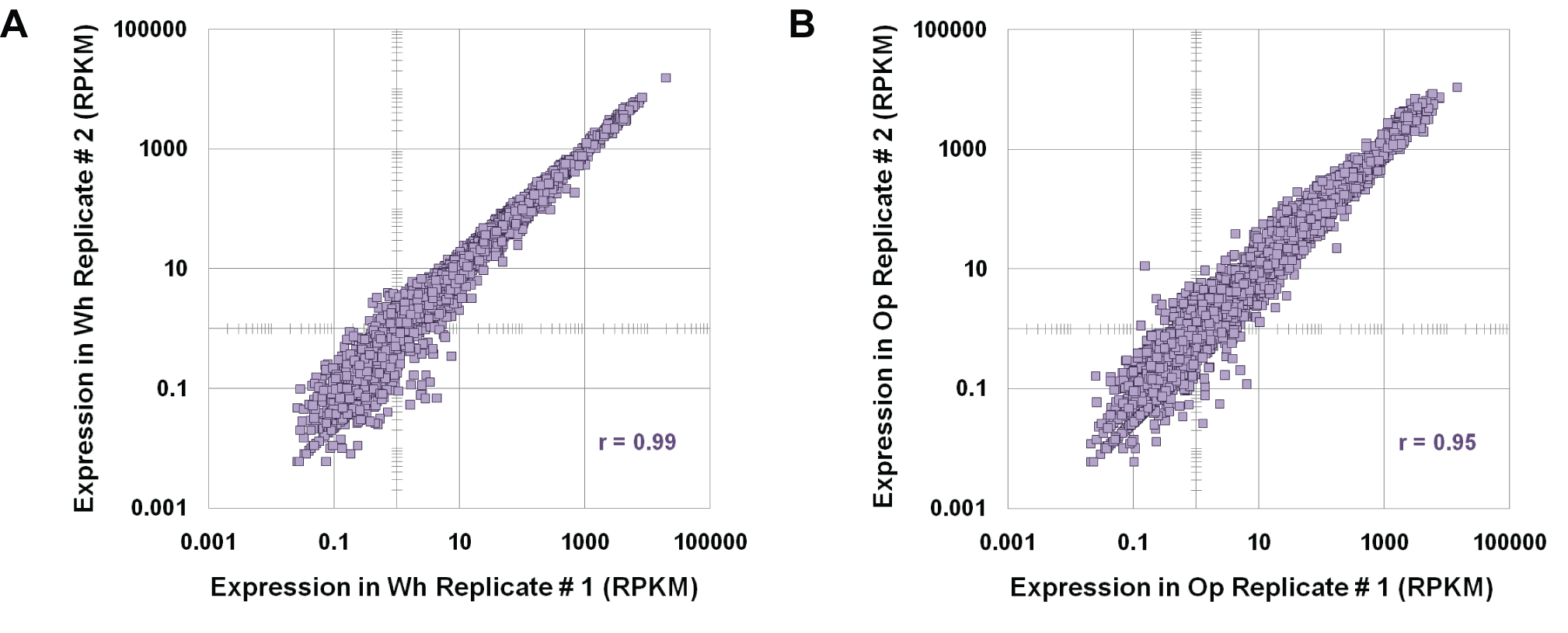

Supplement: Figure S5 — Reproducibility of fold-changes across biological replicates. The abundance of each transcript as estimated by RPKM (reads per kb of transcript per million uniquely aligned reads) from the sequencing of two independently grown (A) white and (B) opaque cell cultures. (0.34 MB TIF) [file pgen.1001070.s005.tif]

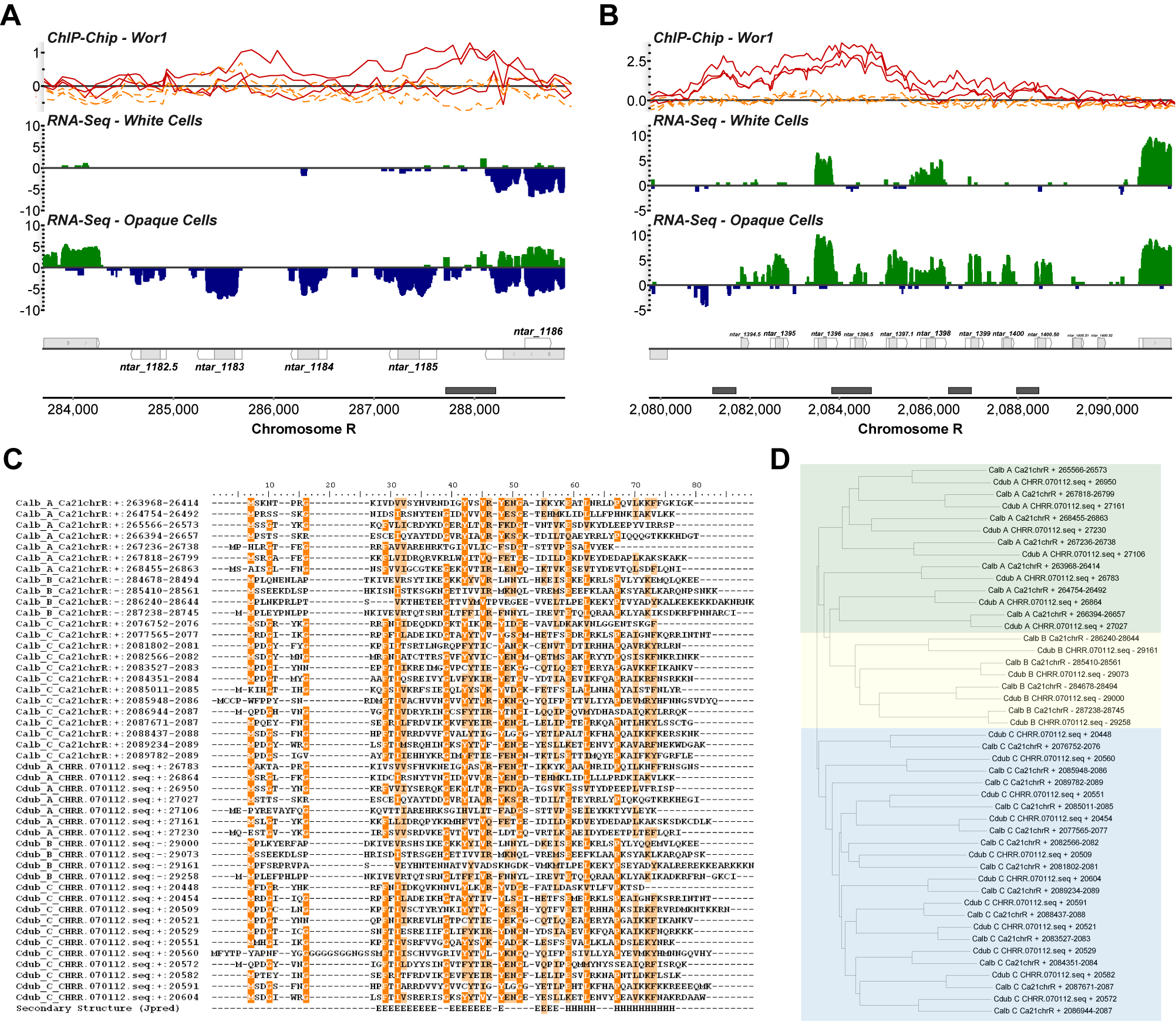

Supplement: Figure S6 — Three clusters of novel Candida-specific ORFs are strongly up-regulated in opaque cells. (A) Expression and Wor1 binding at cluster B of NTAR_1176 homologs on chromosome R (“chrR”). (B) Expression and Wor1 binding at cluster C of NTAR_1176 homologs on chrR. (C) Multiple sequence alignment of all 46 NTAR_1176 homologs found by PSI-BLAST in C. albicans and C. dubliniensis. (D) Neighbor-joining tree of the 46 NTAR_1176 homologs. Clusters A, B, and C are shaded green, yellow, and blue, respectively. (1.23 MB TIF) [file pgen.1001070.s006.tif]

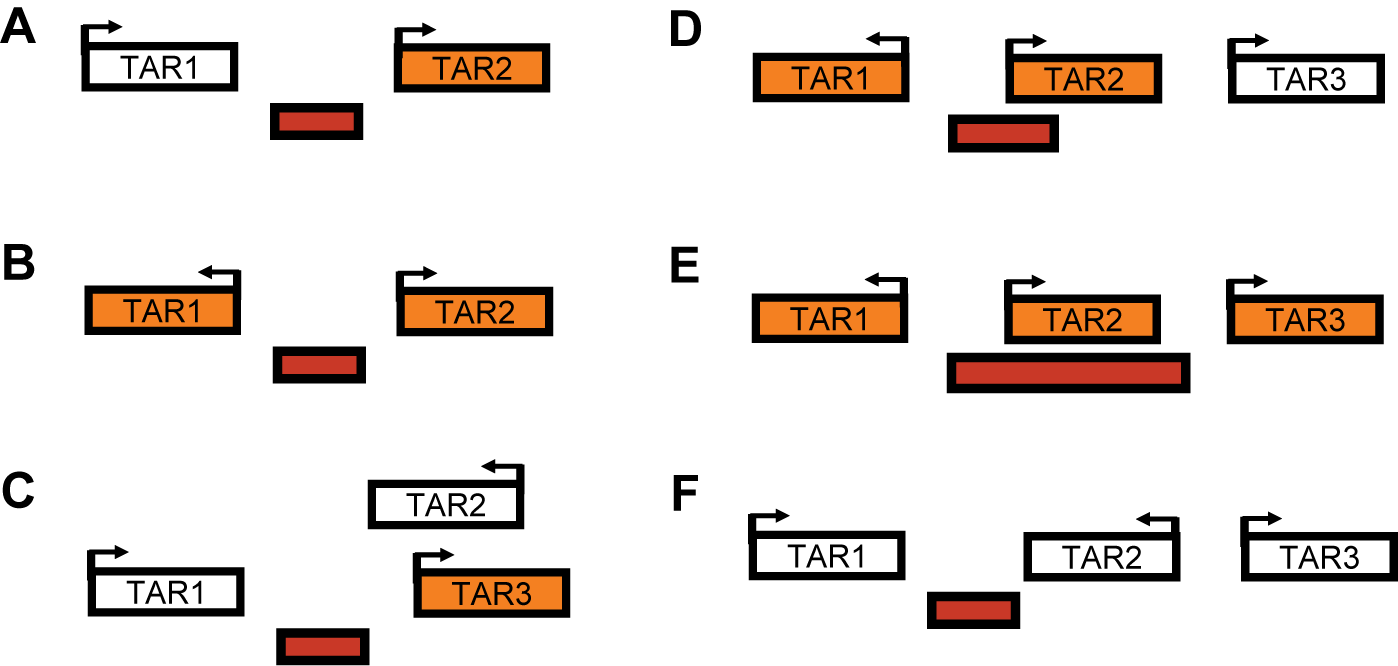

Supplement: Figure S7 — Associating Wor1-bound regions with putatively regulated overlapping and nearby transcripts. Transcripts associated (shaded orange) and not associated (shaded white) with a flanking Wor1-bound region (shaded red) are indicated. Arrows indicate the inferred direction of transcription for each TAR. (0.15 MB TIF) [file pgen.1001070.s007.tif]
